# Diversity of thought: public perceptions of genetic testing across ethnic groups in the UK

**DOI:** 10.1038/s10038-023-01199-1

**Published:** 2023-11-01

**Authors:** Benjamin H. L. Harris, Caitlin McCabe, Hana Shafique, Simon Lammy, Laura Tookman, James Flanagan, Sofia Miron-Barroso, Mark Lythgoe, James Clark, Jason L. Walsh, Matteo Di Giovannantonio, Jonathan Krell

**Affiliations:** 1 Department of Oncology, University of Oxford, Oxford, OX3 7DQ UK; 2 St Anne’s College, 56 Woodstock Rd, Oxford, OX2 6HS UK; 3 Duke University School of Medicine, Durham, NC 27710 USA; 4 Department of Neurosurgery, Institute of Neurological Sciences, Queen Elizabeth University Hospital, Glasgow, G51 4TF UK; 5 Hammersmith Hospital, Imperial College Healthcare NHS trust, London, W12 0HS UK; 6 Department of Surgery and Cancer, Imperial College London, Du Cane Road, London, W12 0HS UK

**Keywords:** Genetics, Social sciences

## Abstract

Genetic testing is becoming rapidly more accessible to the general populous either through or outside healthcare systems. Few large-scale studies have been carried out to gauge public opinion in this growing area. Here, we undertook the largest cross-sectional study on genetic testing in the UK. The primary purpose of this study is to identify the differences in attitudes toward genetic testing across ethnic groups. A cohort of 6500 individuals from a diverse population completed a 72-item survey in a cross-sectional study. Responses between ethnic minority and white individuals in the UK were compared using a wilcoxon rank-sum and chi-square tests. The white cohort was approximately twice as likely to have taken a genetic test and 13% more had heard about genetic testing before the survey. The ethnic minority cohort appeared more apprehensive about the impact of genetic testing on employability. This study highlights that in the UK, significant differences in opinions regarding genetic testing exist between white individuals and ethnic minority individuals. There is an urgent need to develop more inclusive strategies to equally inform individuals from all backgrounds to avoid disparities in the utilisation of genetic testing.

## Introduction

In recent decades, gene sequencing and genetic testing has become increasingly commonplace. What once was a costly process, being performed in top laboratories in the world, can now be purchased directly by patients over the counter and online. Today, there are over 75,000 genetic tests on the market, and about ten new tests entering the market daily [1].

Increased access to genetic testing has the potential to revolutionise disease management, with a greater focus being placed on prevention and individualised treatment. Patients and clinicians can begin to make use of the depth of research that has uncovered the genes and mutations associated with an increased risk for conditions such as heart disease, cancer, and Alzheimer’s disease [2–4]. This can help patients make more informed preemptive healthcare decisions to modify their risk of such diseases, e.g. smoking cessation, diets modification and exercise implementation.

The extent to which individuals take advantage of this new technology can be dependent, not on the scientific validity of the findings, but on patients’ perception and attitude towards genetic testing. Several previous studies have aimed to investigate this. A 2010 study of UK citizens asked participants about their reasons for pursuing personal genome testing and how they would use the information provided about genetic risk [5]. They found that participants were more likely to make healthier lifestyle choices if found to be at a higher genetic risk of a disease. Additionally, they perceived that the test results would be helpful for their children and their doctor [5]. Other studies have been conducted in the USA (*n* =  1041, 31.9% minority groups) [6], Greece (*n* = 1717, ethnicity data of respondents not stated) [7], The Netherlands (*n* = 1795, ethnicity data of respondents not stated but ethnic minorities were said to be “underrepresented”) [8], Belgium (*n* = 1182, ethnicity data of respondents not stated) [9], and the Visegard countries (*n* = 4000, 1000 in each Hungary, Slovakia, Czechia and Poland, ethnicity data of respondents not stated) [10], each aiming to determine public attitudes towards genetic testing and how this can vary. Different demographics were considered, such as gender, marital status and religious beliefs. However, none of the European studies considered the ethnic background of the participants. Thus, there could be a bias in reported results, with the voice of white individuals overshadowing those from ethnic minorities.

This lack of emphasis on patients from ethnic minority backgrounds has been a recurring theme in research and medicine, and has been shown to result in disparities in care and outcomes for these patients [11, 12]. One of the few studies in this area, in 2009, compared responses between non-Hispanic white individuals, Black individuals, and Latino individuals to a questionnaire about genetic testing in the USA (*n* = 1724) [13]. This found that Black individuals and Latino individuals were more likely to face barriers in regard to genetic testing. For instance, Black individuals and Latino individuals were less likely to have insurance coverage for genetic testing and were more likely to feel distrust towards the medical system. Although useful, these findings may not be generalisable to non-insurance-based healthcare systems, e.g. the National Health Service in the UK. Another study from Qatar found generally positive attitudes and willingness to participate in genetic testing (*n* = 837) [14], a finding consistent with the previously mentioned studies from the USA and the Netherlands [6, 8]. However, the major barrier to genetic testing in this study was found to be a lack of time, rather than a lack of information or distrust in the medical system. This demonstrates that the attitudes of individuals from different backgrounds towards genetic testing can vary between countries and needs further investigation.

Research into the role of ethnicity and views towards and participation in genetic studies in the USA yielded themes such as knowledge of genetics as a factor which influences behaviour [15]. Work of this nature is needed in the UK. Determining whether there is a difference in opinions between individuals from ethnic minority backgrounds in European countries, and, if so, where these differences occur, will be vital not only to predict the future applicability of genetic testing, but also in identifying barriers. If there are any disparities emerging in the use or knowledge surrounding genetic testing, they can be identified and addressed.

## Methods

### Study design, setting, and population

The survey was created by researchers (genetics and cancer specialists) at Imperial College London in collaboration with patient representatives. Survey questions are found in Table S1. The aim of the study was to assess societal opinions on genetic testing. Before the survey was conducted, ethical approval was obtained (Research Ethics Committee [REC] reference number: 18/EM/0070I) and leading observational study reporting guidelines were noted [16]. The survey was distributed to patients aged 18 years and above who were registered within London and the surrounding area. Each participant completed a consent form prior to filling out the survey. Participants were recruited through (a) an email link distributed by general practitioners (GPs), (b) a link on a social media post on Facebook by the marketing agency nativve (https://www.nativve.com/) or (c) by being asked to complete the questionnaire whilst visiting their GP practice. The questionnaire was distributed electronically and completed independently, though patients were encouraged to discuss it with friends, family, or their GP if necessary. The aim was to collect at least 5000 patients over an 18-month period (March 2019 to December 2021).

### Measurements

The anonymised questionnaire was delivered to participants via an online platform. Each participant was asked for demographic information: ethnic group, gender, marital status and religious beliefs. The 72-item survey covered five themes: (1) *knowledge and familiarity with genetic testing [12 items]*, (2) *actions or feelings as a result of a supposed genetic test [14 items]*, (3) *concerns or apprehensions surrounding genetic tests [6 items]*, (4) *predicted future applications of genetic testing [18 items]*, (5) *personal understanding of biology or genetics [17 items]* as well as collecting *demographic information [5 items]*. Participants had slightly different response types across the sections:

- *Knowledge and familiarity with genetic testing*: The questions in this section were answered with: yes, unsure or no.
- *Actions or feelings as a result of a supposed genetic test*: the majority of items in this section were answered on a 6-point Likert scale, in which 1 corresponded to strong disagreement with the statement and 6 indicated strong agreement. Four questions were answered as a percentage of their likelihood to take the described action or change. Respondents could select from the following: Never, <10%, 10–30%, 30–50%, 50–80%, or 80–100%.
- *Concerns or apprehensions surrounding genetic tests, Personal understanding of biology or genetics & Predicted future applications of genetic testing*: all items in these sections were answered on a 6-point Likert scale, with 1 indicating strong disagreement and 6 strong agreement.

### Statistical analysis

Analyses were carried out using Python software, version 3.7 (available from www.python.org). The respondents were compared as two groups based on historical majorities of indigenousness in the UK: ethnic minority (Group EM+) and white (Group W), detailed in Table 1. Subcategories within these groups originated from the UK Office for National Statistics and ethnic categories used in the UK National Census. We opted to use the term ethnic minority, which incorporates Black individuals, Asian individuals and other minority groups, because of the UK government’s incorporation of the acronym BAME in 2021 causing disquiet amongst ethnic majority and minority groups. We acknowledge there is no term which can accurately encompass the highly disparate racial, cultural and religious entities that comprise ethnic minority groups. The authors are from a diverse range of backgrounds and have opted to use the term ethnic minority as it is established, understandable and least likely to cause offence. All ethnic minorities are grouped together due to the numbers being too small across subgroups to permit accurate data analysis. This approach has been used in previous primary studies and systematic reviews across several different fields. Further, there is uncertainty on where individuals with combined ethnic backgrounds should fall (e.g. “Mixed White & Black”). Here we include individuals such as this within the EM+ group.

**Table 1:** Subgroups within the ethnic minority and white cohorts

Cohort name	Subgroup name	Count
Group EM+ (Ethnic Minority)	**Black, Black British, Caribbean or African**	
	Caribbean	111
	African	162
	Any Other Black Background	224
	**Asian or Asian British**	
	Indian	341
	Pakistani	110
	Bangladeshi	26
	Chinese	74
	Any Other Asian Background	297
	**Mixed or multiple ethnic groups**	
	Mixed White & Black	76
	Mixed White & Asian	75
	Any Other Mixed Background	131
	**Other ethnic group**	
	Any Other Ethnic Group	186
Group W (White)	**White**	
	British	3113
	Irish	328
	Any Other White Background	1055

Detailed breakdown of the ethnic subgroups within the ethnic minority (Group EM+) and white (Group W) cohorts

The two groups were compared using a chi-squared test for nominal variables (as in previous work [8]) and Wilcoxon rank-sum test was used to evaluate ordinal variables. A Bonferroni corrected *p*-value of *p* < 6.94E−4 was considered statistically significant (threshold defined as 0.05/72 with 72 corresponding to the number of items analysed from the survey). A *p*-value below this corrected threshold denotes a significant difference in opinion between the two groups. We highlight the percentage in agreement (somewhat agree, agree, and strongly agree) in each cohort, and its significance, in the text. Note the chi-squared test compares expected and observed values for individuals in each group, therefore where relevant we include percentages in all the categories to better illustrate significant differences between the groups.

## Results

### Summary of participants

**Fig. 1:**
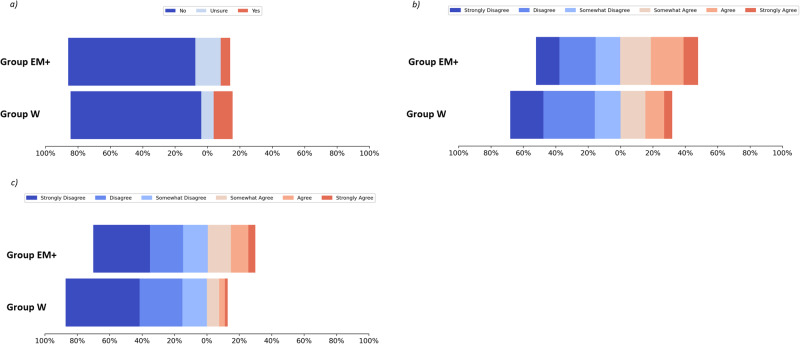
Statements perceived and questions answered most differently between Group EM+ and Group W in three sections of the survey. The statements perceived and questions answered most differently in the first three sections of the survey: **a** knowledge and familiarity with genetic testing (“I have previously undertaken a genetic test”), **b** actions or feelings as a result of a supposed genetic test (“If I learnt from a genetic test that I had an increased risk of developing cancer, I would worry that it would affect my chances of finding a job”), **c** predicted future applications of genetic testing (“In 5–10 years time, potential employees will have to do a genetic test before they are hired”) and there were no questions answered significantly differently between the two groups in “concerns or apprehensions surrounding genetic testing”. Each bar sums to 100% and the distribution of each category is shown using different colours. For (**a**) red = yes, light blue = unsure and dark blue = no, whereas in (**b**–**c**) light to dark red denotes agreement and light to dark blue denotes disagreement

The survey was completed by 6500 participants. Of these, 191 had missing ethnicity data. Within the remaining 6309 participants, 1813 individuals were in the EM+ group and 4496 in Group W (Table 1). The two cohorts did not differ significantly in terms of gender, but were significantly different for marital status, religion, and the impact of religion on their beliefs (Table 2 and residuals in Table S2). Results of all questions are summarised in Appendix 1 and Table S3. The statements perceived and questions answered most differently between Group EM+ and Group W in four sections are summarised in Fig. 1.

**Table 2:** Characteristics of participants in the ethnic minority and white cohorts

	Whole *n* = 6500 (*n* = %)	Group EM+ (Ethnic Minorities) *n* = 1813 (*n* = %)	Group W (White) *n* = 4496 (*n* = %)	Missing ethnicity *n* = 191 (*n* = %)	*P*-value: Group EM+ vs. Group W
Gender					*p* = 0.131
Female	4260 (65.54%)	1164 (64.20%)	2978 (66.24%)	118 (61.78%)	
Male	2240 (34.46%)	649 (35.80%)	1518 (33.76%)	73 (38.22%)	
Marital status					*p* < 6.49E−4
Married	3040 (46.77%)	857 (47.27%)	2103 (46.77%)	80 (41.89%)	
Cohabiting	827 (12.72%)	179 (9.87%)	628 (13.97%)	20 (10.47%)	
Separated/ Divorced/Widowed	889 (13.68%)	181 (9.98%)	682 (15.17%)	26 (13.61%)	
Single/Never married	1588 (24.43%)	548 (30.23%)	1002 (22.29%)	38 (19.89%)	
Other	156 (2.40%)	48 (2.65%)	81 (1.80%)	27 (14.14%)	
Religion					*p* < 6.49E−4
No religion	2300 (35.39%)	370 (20.41%)	1895 (42.15%)	35 (18.32%)	
Catholic	1085 (16.69%)	200 (11.03%)	867 (19.29%)	18 (9.42%)	
Christian (Other)	1623 (24.97%)	357 (19.69%)	1247 (27.73%)	19 (9.95%)	
Jewish	121 (1.86%)	8 (0.43%)	108 (2.39%)	5 (2.62%)	
Muslim	376 (5.78%)	290 (16.00%)	59 (1.31%)	27 (14.14%)	
Hindu	295 (4.54%)	287 (15.83%)	2 (0.05%)	6 (3.14%)	
Buddhist	74 (1.14%)	38 (2.10%)	32 (0.72%)	4 (2.10%)	
Other	284 (4.37%)	166 (9.16%)	107 (2.38%)	11 (5.76%)	
Rather not say	342 (5.26%)	97 (5.35%)	179 (3.98%)	66 (34.55%)	
Religious impact on decisions					*p* < 6.49E−4
Very important	614 (9.45%)	338 (18.64%)	248 (5.52%)	28 (14.66%)	
Somewhat important	717 (11.03%)	290 (16.00%)	405 (9.01%)	22 (11.52%)	
Only a little important	668 (10.28%)	212 (11.69%)	448 (9.96%)	8 (4.19%)	
Not at all important	3907 (60.11%)	777 (42.86%)	3068 (68.24%)	62 (32.46%)	
Rather not say	594 (9.13%)	196 (10.81%)	327 (7.27%)	71 (37.17%)	

Details of the genders, marital status, religious beliefs and the perceived impact of religious beliefs on decisions in ethnic minority (Group EM+) and white (Group W) individuals

### Knowledge and familiarity with genetic testing

Respondents were asked if they had heard about genetic testing, if they had undertaken a genetic test or had been diagnosed with an inherited genetic condition. As a whole, participants were fairly familiar with genetic testing, with 69.94% of them having heard about genetic testing prior to taking this survey. Additionally, 10.09% had undertaken a genetic test and 6.86% had been diagnosed with an inherited genetic condition.

Group W tended to be more familiar with genetic testing, with about 13% more having heard about genetic testing as compared to Group EM+ (Group EM+ = 61.00%, Group W = 73.98%; *p* < 6.94E−4). Additionally, those in Group W were almost twice as likely to have taken a genetic test compared to Group EM+ (Group EM+ = 5.90%, Group W = 11.65%; *p* < 6.94E−4). This perhaps partially explained the results showing that a slightly increased proportion of white individuals had been (a) diagnosed with an inherited genetic condition (Group EM + = 6.78% Yes, 22.06% Unsure, 71.16% No ; Group W = 6.85% Yes, 14.02% Unsure, 79.13% No, *p* < 6.94E-4), (b) found to carry an inherited genetic abnormality linked to an increased risk of developing cancer (Group EM+ = 1.88% Yes, 25.37% Unsure, 72.75% No; Group W = 2.07% Yes, 14.46% Unsure, 83.47% No, *p* < 6.94E−4) and (c) been found to carry an inherited genetic abnormality that is linked to an increased risk of developing a specific disease (Group EM+ = 4.91% Yes, 25.92% Unsure, 69.17% No; Group W = 5.52% Yes, 15.24% Unsure, 79.25% No, *p* < 6.94E−4). The EM+ group reported having more relatives who carry an inherited genetic abnormality that has been linked with an increased risk of developing a cancer (Group EM + = 7.73% Yes, 33.75% Unsure, 58.52% No; Group W = 7.22% Yes, 26.45% Unsure, 66.33% No, *p* < 6.94E−4).

### Actions or feelings as a result of genetic test

Respondents were asked how a genetic test might affect them, as well as how much they would trust the test results. As a whole, participants were interested in taking a genetic test to see if they had increased risk for cancer, and mostly felt that a genetic test would help them plan for the future (86.06% agreed), but they were worried how it would affect their health and/or life insurance (73.81% agreed).

Those in the EM+ group tended to showed concern around the future implications of genetic results to their livelihoods. When asked *“if I learnt from a genetic test that I had an increased risk of developing cancer, I would worry that it would affect my chances of finding a job*”, EM+ participants were more likely to agree (Group EM+: 48.09% agreed; Group W: 31.90% agreed, *p* < 6.94E−4). Perhaps this reflects a more general underlying anxiety about the use of genetic testing for minority populations. There have been a number of instances of compulsory DNA sample collection in various countries for surveillance and control programmes [17], some of these practices have been thought to be discriminatory in nature. There was no significant difference between the two groups in their concern about how a genetic test would affect their health and/or life insurance or that the statement *“treatment and prevention options for cancer are limited, so learning from a genetic test that I had an increased risk of developing cancer wouldn’t help much*”. However, the EM+ group were more inclined to plan for the future if they learnt that they had an increased risk of developing cancer (Group EM+: 87.98% agreed; Group W: 85.63% agreed, *p* < 6.94E−4). Further, there were no significant differences between Group EM+ and Group W in their concern about their own, their partner’s or their family’s emotional responses.

The two groups differed significantly when asked at what level of risk they would initiate medication to modify their risk of cancer. A lifetime risk of cancer of 30–50% in Group EM+ was selected most often for when to start a new medication (23.17% of respondents) whereas Group W most selected 50–80% (25.33% of respondents). Both groups most frequently chose a 10–30% lifetime risk of developing cancer to prompt them to make lifestyle or diet changes (Group EM+: lifestyle—24.60%, diet—25.92%; Group W: lifestyle—28.54%, diet—30.43%). Finally, 50–80% lifetime risk was most often selected by both groups to undergo preventative surgery (Group EM+: 28.13%; Group W: 31.83%).

### Concerns or apprehensions surrounding genetic testing

Overall, the respondents were moderately concerned that a genetic test would change their future (57.41% agreed) and respondents were fairly undecided on the subject of not wanting to be tested for specific diseases. There was no significant difference between groups with regard to increased concern or apprehension on how a genetic test might affect their future or what diseases for which they might have shown increased risk. Of the four statements around apprehensions towards genetic tests, Group EM+ were likely to agree with all of them, but these differences were not statistically significant under Bonferroni adjustment: (a) “*I do not want to know what kind of diseases I could get in the future*” (Group EM+: 25.97% agreed; Group W: 23.90% agreed), (b) “*The idea of a genetic test frightens me*” (Group EM+: 41.20% agreed; Group W: 35.85%), (c) “*I worry that having a genetic test might change my future*” (Group EM+: 65.19% agreed; Group W: 54.33% agreed) and (d) *“It would be too upsetting to learn from a genetic test that I have an increased risk of developing cancer, so I am happier not knowing”* (Group EM+: 24.16% agreed; Group W: 20.88% agreed).

### Predicted future applications of genetic testing

Participants overall were strongly in favour of increasing funding for genetic tests (94.14% agreed), making them more available (93.24% agreed) and using genetic tests in the recruitment of patients for cancer screening programmes (84.31% agreed). Additionally, respondents wanted test results to be confidential (95.96% agreed), and saw them as a positive advancement in treating disease (97.05% agreed). Participants did not have strong predictions for the future applications of genetic testing, although they did not think that test results would be used routinely in employment (82.32% disagreed with the statement “*In 5–10 years time, potential employees will have to do a genetic test before they are hired*”).

Participants were also asked about access to the information from genetic tests. Group W were slightly more in favour of the results remaining confidential (Group EM+: 95.36% agreed; Group W: 96.26% agreed, *p* < 6.94E−4). However, Group EM+ thought individuals should legally have to inform relatives about their test results (Group EM+: 43.51% agreed; Group W: 27.73% agreed, *p* < 6.94E−4).

The two groups were not significantly different in their opinions that (a) people should have more genetic tests, (b) they would consider having a genetic test that was available over the counter at a chemist or supermarket (without the involvement of a healthcare professional), (c) genetic tests should be used to select people for cancer screening programs and (d) their interest in finding out if a disease had been inherited (both groups overall were in markedly favour). Further, both groups were overwhelmingly supportive of increasing funding for research (Group EM+: 93.93% agreed; Group W: 94.51% agreed).

Participants were also asked about how they envisioned the uses of genetic testing changing in 5–10 years. Both groups were similarly unsure on the existence of genetic passports. However, Group EM+ were more likely to agree that (a) genetic testing would be used by employers for selecting new hires (Group EM+: 29.34% agreed, Group W: 12.83% agreed, *p* < 6.94E−4) and (b) genetic testing would be utilised by insurance companies setting premiums (Group EM + : 46.05% agreed; Group W: 35.92% agreed, *p* < 6.94E−4).

### Personal understanding of biology or genetics

**Fig. 2:**
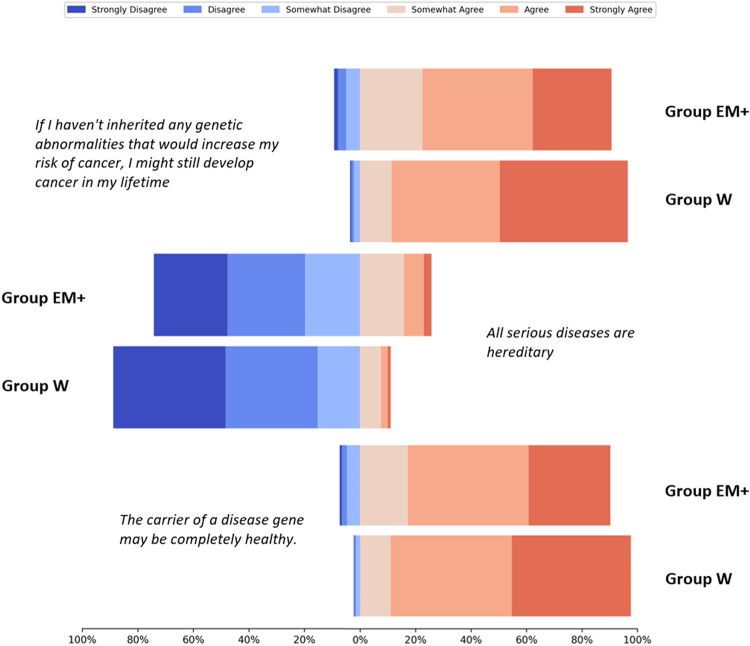
Statements perceived most differently between Group EM+ and Group W in their in their personal understanding of biology and genetics. The Likert scale chart shows marked differences between the two groups. The chart represents the items with the three lowest *p*-values (greatest difference between the groups). Each bar sums to 100% and the distribution of each category is shown using different colours (light to dark red denotes agreement whereas light to dark blue denotes disagreement)

Out of a series of 17 questions related to biology and genetics, the responses of Group EM+ and Group W significantly differed in number of these. As a whole, the respondents tended to choose the correct answer, however, Group EM+ tended to be less confident in their answers (shown in Fig. 2).

Both populations had similar opinions, both overwhelmingly in favour that genes: (a) are a piece of DNA (b) part of a chromosome (c) are inside cells (d) come in pairs; one copy from each parent and (e) that a person has ~22,000 genes. Overall, this highlights a similar baseline understanding of genetics in both groups. However, Group W seemed perhaps more aware of biological nuances. For instance, the onset of certain diseases is due to a combination of genes, environment, and lifestyle (Group EM+: 93.93% agreed; Group W: 96.00% agreed, *p* < 6.94E−4). Given the increasing importance of genomic testing in clinical care, this illuminates an important consideration about examining the way individuals understand and communicate about genetic information. The implications of these findings are aligned with other studies suggesting that genomics-related health literacy domains (i.e. knowledge, understanding) should inform educational programmes for genomic information [18].

There was some confusion over the heritability of disease, although overall understanding was high. A larger proportion of Group EM+ agreed with the notion that all serious diseases are hereditary (Group EM+: 25.81%% agreed; Group W: 11.14% agreed, *p* < 6.94E-4) and were more inclined to believe that the child of an individual with a genetic disease *always* inherits the gene causing the disease (Group EM+: 50.47% agreed; Group W: 32.25% agreed, *p* < 6.94E−4). A smaller proportion of Group EM+ agreed with the statement *“If I have inherited genetic abnormalities that increase my risk of cancer, this does not mean that I will definitely develop cancer”* (Group EM + : 89.46% agreed; Group W: 93.26% agreed, *p* < 6.94E-4) and *“The carrier of a disease gene may be completely healthy”* (Group EM + : 92.55% agreed; Group W: 97.64% agreed, *p* < 6.94E-4).

## Discussion

The lack of emphasis on patients from ethnic minority backgrounds has resulted in disparities in care and outcomes for patients. Therefore, we encouraged participants to disclose their ethnic background. Of the 6500 participants, 4496 individuals identified as white, 1813 ethnic minority and 191 did not disclose their ethnicity. Overall, participants were fairly familiar with genetic testing, with ~70% having heard about it prior to the survey and around 1 in 10 having undertaken a genetic test themselves. In 2010, a UK study showed only 13% of respondents were aware of genetic tests [5]. It was clear that participants were overwhelmingly in favour of increasing funding for genetic testing and making it more available (94% and 93% in agreement, respectively). These feelings echo those described in other studies [6, 8]. About three quarters of individuals worried about how genetic tests would affect their health and/or life insurance (73.81% agreed), despite most residents of the UK not paying for private health insurance.

A major insight from this descriptive study comes from individuals from ethnic minority backgrounds (Group EM+). Group EM+ made up around one quarter of individuals and had a similar proportion of women to the white group. There was a difference in religious persuasions in this group with Islam and Hinduism being more common. Overall, Group EM+ appeared less familiar with genetic testing. Group EM+ were significantly less likely to have heard about genetic testing (61% vs. 74%) and were approximately half as likely to have taken a genetic test (6% vs. 12%). One reason for this might be individuals in Group EM+ were significantly more likely to have been refused a genetic test by a doctor or genetics specialist. A study in the USA found that ethnic minority individuals were less likely to receive a genetic test due to lack of knowledge, health insurance coverage, and a distrust in the medical system [13]. Those who identified as Black or Latino were more likely to distrust their medical doctor in keeping their clinical information private. Distrust in medical practitioners was not overtly obvious in this study, however, Group EM+ did consider that genetic testing might influence employment. However public policy, as yet, heirs in the main on the side of the individual with consent having to be given before data can be utilised by employers [19]. Educating EM+ communities on this point might be beneficial. It is important to consider the fact that the low rate of genetic testing in the EM+ group is likely multifactorial. Another USA study revealed those with higher education level and higher household income had an overall greater interest in genetic testing [20]. Although we did not directly assess factors as such, we acknowledge the potential patterning they have on our findings and perhaps might be interesting to explore in future UK studies.

This study revealed a baseline understanding of genetics, particularly that genes are made of DNA, come in pairs, and are inside of cells. However, there was some confusion regarding the heritability of disease. One remarkable finding was that a quarter of Group EM+ and ~1 in 10 of Group W believed that all serious diseases are hereditary. The relative lack of uptake of genetic testing in the EM+ group perhaps illustrates how the fear of testing outweighs views of potential benefits. Prospectively trialling genetics educational interventions in communities and testing their influence on genetic testing perceptions would be interesting in future studies.

Although having some major positives, including a large sample size and diversity data, this work does have some important limitations. Firstly, this study was undertaken predominantly in London and the surrounding area, and this may not be representative of the entirety of the UK or beyond. Information on the age of participants and their occupation was not collected. As such, it is likely, as is the case for many volunteer study cohorts, that there may be a higher representation of the middle and higher social classes than the national average [5]. We also appreciate certain factors that tend to be closely linked, such as ethnicity and religion, so rather than trying to artificially untangle these we have stated the results descriptively. Further, despite the large sample size there were not enough participants from different ethnic groups to draw reliable conclusions on the viewpoints of individual ethnicities.

In conclusion, this study highlights that the UK public are highly in favour of genetic testing with 1 in 10 individuals having already undertaken some form of genetic testing. We find significant differences exist between white and ethnic minority attitudes towards genetic testing, with ethnic minority individuals tending to be less informed and more concerned about the employment repercussions of genetic tests. These descriptive findings could be used to inform future educational and genetic testing initiatives in specific communities.

### Supplementary information

Table S1

Table S2

Table S3

Appendix 1

## Data Availability

Data are available upon request.

## References

[1] Phillips KA, Deverka PA, Hooker GW, Douglas MP (2018). Genetic test availability and spending: where are we now? Where are we going?. Health Aff.

[2] Institute of Medicine (US) Committee on Assessing Genetic Risks, Andrews, LB, Fullarton, JE, Holtzman, NA & Motulsky, AG Genetic Testing and Assessment. Assessing genetic risks: implications for health and social policy. USA: National Academies Press; 1994. 25144102

[3] Di Giovannantonio M, Harris BH, Zhang P, Kitchen-Smith I, Xiong L, Sahgal N (2021). Heritable genetic variants in key cancer genes link cancer risk with anthropometric traits. J Med Genet.

[4] Bloss CS, Jeste DV, Schork NJ (2011). Genomics for disease treatment and prevention. Psychiatr Clin N Am.

[5] Cherkas LF, Harris JM, Levinson E, Spector TD, Prainsack B (2010). A survey of UK public interest in internet-based personal genome testing. PLoS ONE.

[6] Kerath SM, Klein G, Kern M, Shapira I, Witthuhn J, Norohna N (2013). Beliefs and attitudes towards participating in genetic research - a population based cross-sectional study. BMC Public Health.

[7] Mai, Y, T Koromila, A Sagia, DN Cooper, G Vlachopoulos, G Lagoumintzis, et al. A critical view of the general public’s awareness and physicians’ opinion of the trends and potential pitfalls of genetic testing in Greece. Per Med. 2011;8:551–61. 10.2217/pme.11.48. 10.2217/pme.11.48 29793257

[8] Henneman L, Vermeulen E, van El CG, Claassen L, Timmermans DR, Cornel MC (2012). Public attitudes towards genetic testing revisited: comparing opinions between 2002 and 2010. Eur J Hum Genet..

[9] Chokoshvili D, Belmans C, Poncelet R, Sanders S, Vaes D, Vears D (2017). Public views on genetics and genetic testing: a survey of the general public in Belgium. Genet Test Mol Biomark.

[10] Bíró K, Dombrádi V, Fekete Z, Bányai G, Boruzs K, Nagy A, Ádány R (2020). Investigating the knowledge of and public attitudes towards genetic testing within the Visegrad countries: a cross-sectional study. BMC Public Health.

[11] Esegbona-Adeigbe S (2021). The impact of a Eurocentric curriculum on racial disparities in maternal health. Eur J Midwifery.

[12] Martin AR, Kanai M, Kamatani Y, Okada Y, Neale BM, Daly MJ (2019). Clinical use of current polygenic risk scores may exacerbate health disparities. Nat Genet..

[13] Suther S, Kiros G-E (2009). Barriers to the use of genetic testing: a study of racial and ethnic disparities. Genet Med.

[14] Abdul Rahim HF, Ismail SI, Hassan A, Fadl T, Khaled SM, Shockley B (2020). Willingness to participate in genome testing: a survey of public attitudes from Qatar. J Hum Genet..

[15] Fisher ER, Pratt R, Esch R, Kocher M, Wilson K, Lee W, Zierhut HA (2020). The role of race and ethnicity in views toward and participation in genetic studies and precision medicine research in the United States: a systematic review of qualitative and quantitative studies. Mol Genet Genom Med.

[16] von Elm E, Altman DG, Egger M, Pocock SJ, Gøtzsche PC, Vandenbroucke JP, STROBE I (2007). Strengthening the Reporting of Observational Studies in Epidemiology (STROBE) statement: guidelines for reporting observational studies. BMJ.

[17] Forzano F, Genuardi M, Moreau Y, European Society of Human Genetics. (2021). ESHG warns against misuses of genetic tests and biobanks for discrimination purposes. Eur J Hum Genet..

[18] Kaphingst KA, Blanchard M, Milam L, Pokharel M, Elrick A, Goodman MS (2016). Relationships between health literacy and genomics-related knowledge, self-efficacy, perceived importance, and communication in a medically underserved population. J Health Commun..

[19] Geppert CMA, Roberts LW (2005). Ethical issues in the use of genetic information in the workplace: a review of recent developments. Curr Opin Psychiatry.

[20] Dusic EJ, Bowen DJ, Bennett R, Cain KC, Theoryn T, Velasquez M (2022). Socioeconomic Status and Interest in Genetic Testing in a US-Based Sample. Healthcare.

