# Supplementary material for: Diversity of thought: public perceptions of genetic testing across ethnic groups in the UK
Source: J Hum Genet. 2023 Nov 1;69(1):19–25. doi: 10.1038/s10038-023-01199-1 (PMC10774120; doi:10.1038/s10038-023-01199-1)
Supplement: Supplementary file 2 — Table S2 [file 10038_2023_1199_MOESM2_ESM.docx]

|  | **Group EM+ (Ethnic Minorities)** n = 1813 (n = %) | **Group W (White)** n = 4496 (n = %) |
| --- | --- | --- |
| **Gender** |  |  |
| *Female* | -34.48 | -54.32 |
| *Male* | -24.94 | -39.29 |
| **Marital Status** |  |  |
| *Married* | -29.15 | -45.92 |
| *Cohabiting* | -15.22 | -23.98 |
| *Separated/ Divorced/Widowed* | -15.74 | 24.79 |
| *Single/Never Married* | -21.09 | -33.23 |
| *Other* | -6.08 | -9.59 |
| **Religion** |  |  |
| *No Religion* | -25.50 | -40.17 |
| *Catholic* | -17.50 | -27.57 |
| *Christian (Other)* | -21.46 | -33.80 |
| *Jewish* | -5.77 | -9.09 |
| *Muslim* | -10.00 | -15.77 |
| *Hindu* | -9.10 | -14.35 |
| *Buddhist* | -4.48 | -7.06 |
| *Other* | -8.85 | -13.95 |
| *Rather Not Say* | -8.90 | -14.02 |
| ***Religious Impact on Decisions*** |  |  |
| *Very Important* | -12.96 | -20.43 |
| *Somewhat Important* | -14.12 | -22.25 |
| *Only a Little Important* | -13.76 | -21.68 |
| *Not at All Important* | -33.23 | -52.33 |
| *Rather Not Say* | -12.25 | -19.30 |
