# Supplementary material for: Diversity of thought: public perceptions of genetic testing across ethnic groups in the UK
Source: J Hum Genet. 2023 Nov 1;69(1):19–25. doi: 10.1038/s10038-023-01199-1 (PMC10774120; doi:10.1038/s10038-023-01199-1)

**Knowledge of genetic testing**

- *I had heard about genetic testing before this questionnaire.*


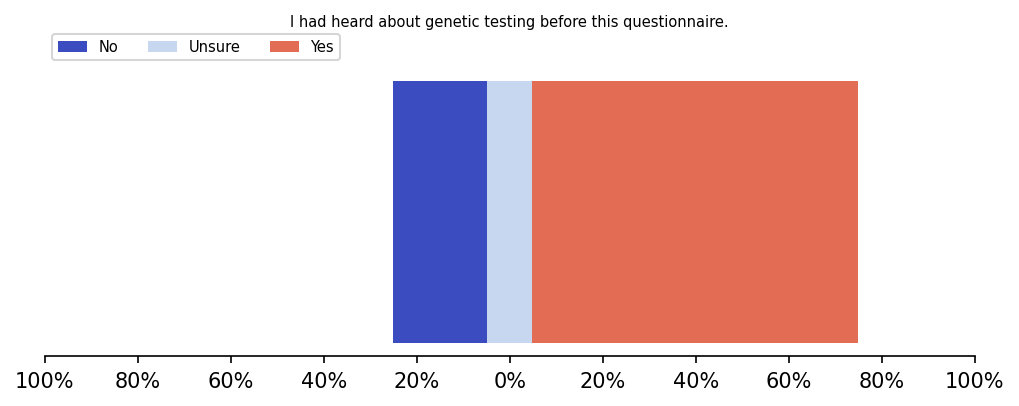


- *I have previously undertaken a genetic test.*


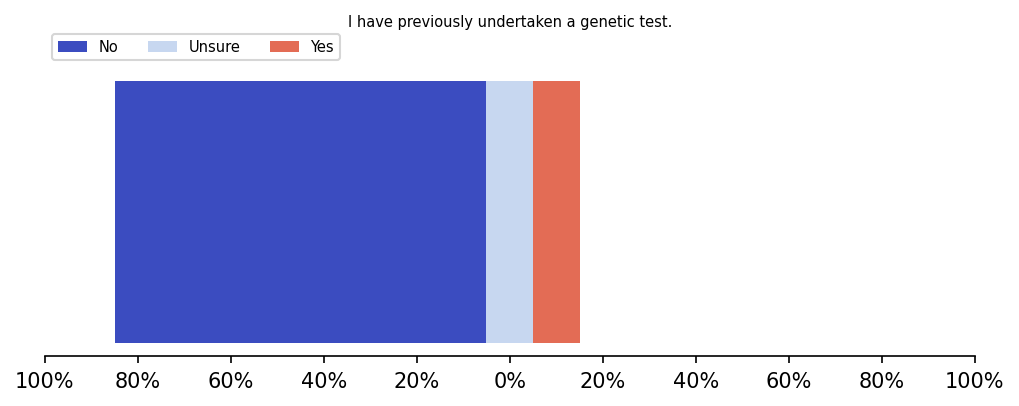


- *I have previously been diagnosed with an inherited genetic condition.*


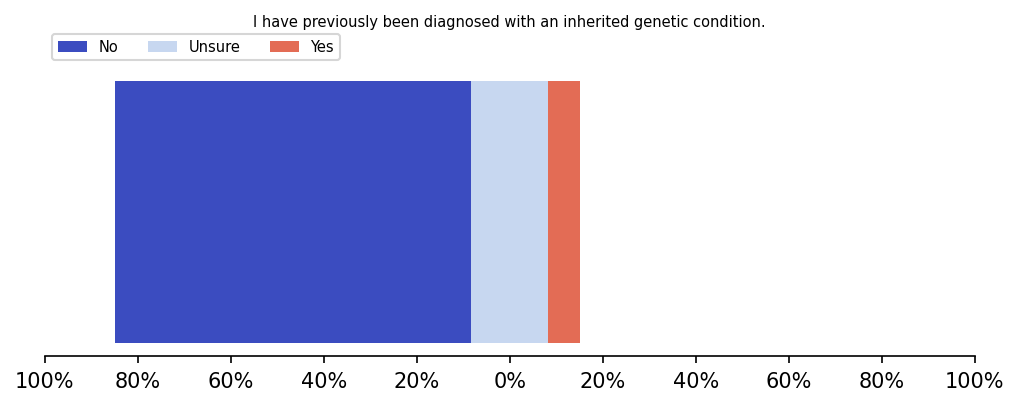


- *I have previously been found to carry an inherited genetic abnormality that has been linked with an increased risk of developing a specific disease.*


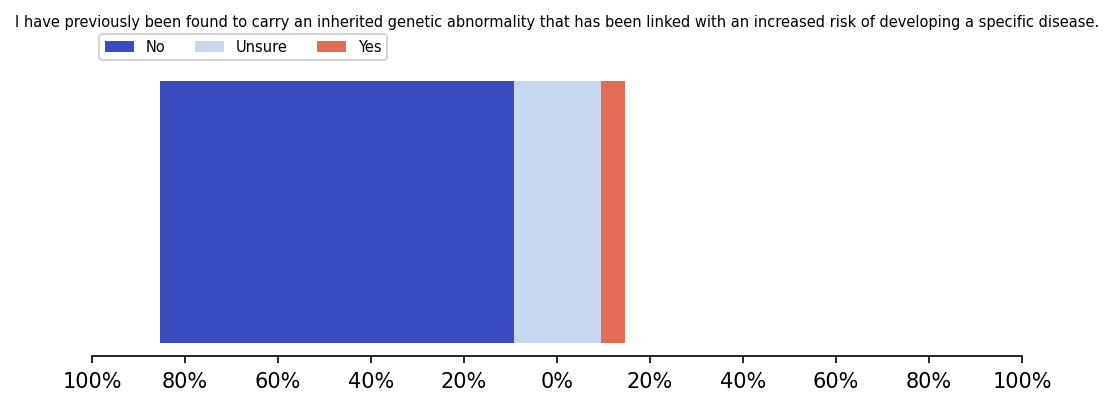

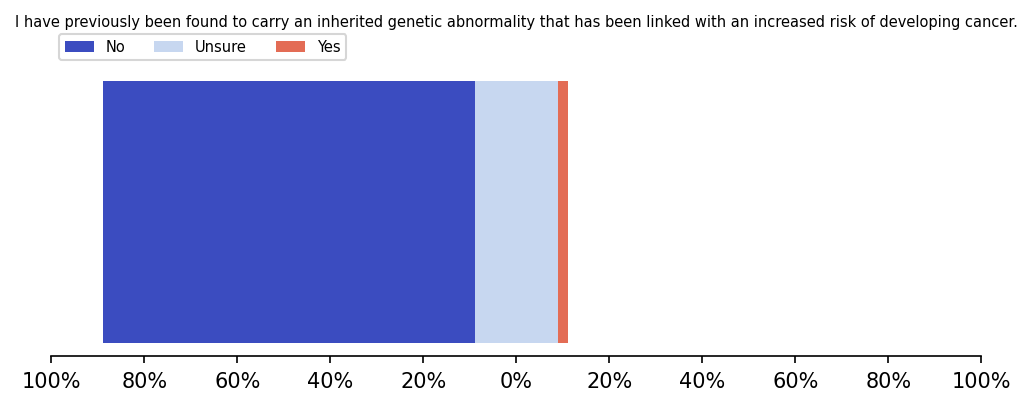


- *I have previously been found to carry an inherited genetic abnormality that has been linked with an increased risk of developing cancer.*


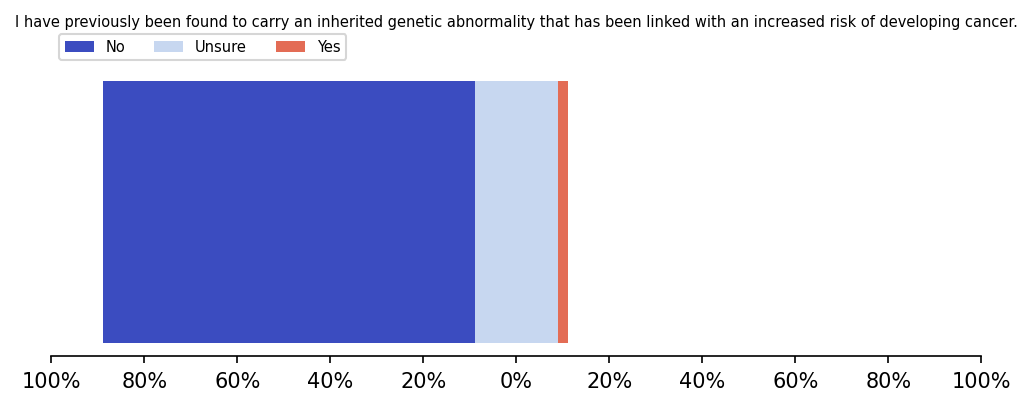


- *I have a relative who has previously been diagnosed with an inherited genetic condition.*


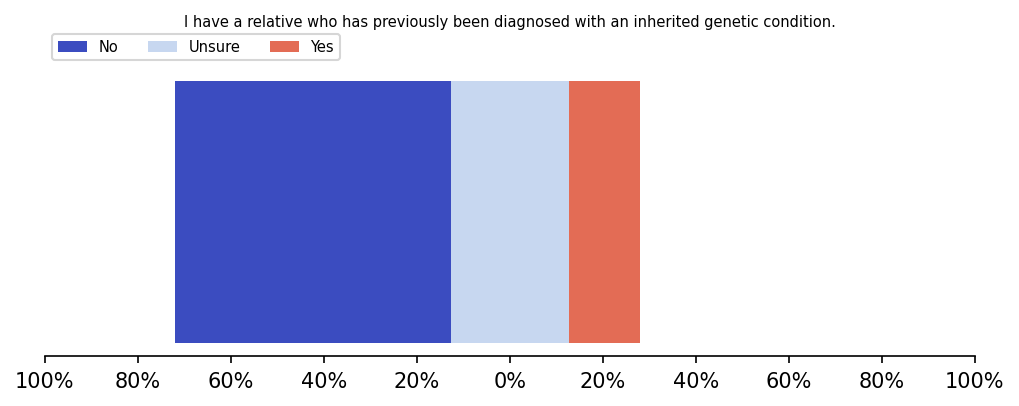


- *I have a relative who carries an inherited genetic abnormality that has been linked with an increased risk of developing a cancer.*


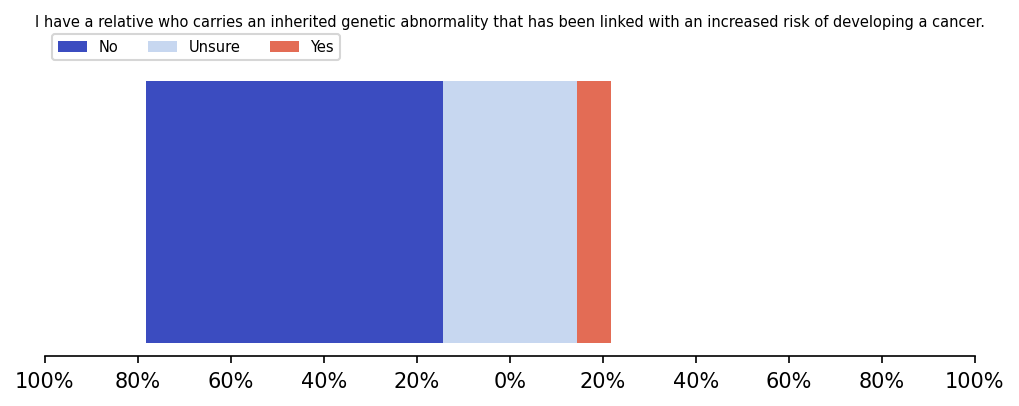


- *I believe that cancer screening programs should be made available to larger groups of people.*


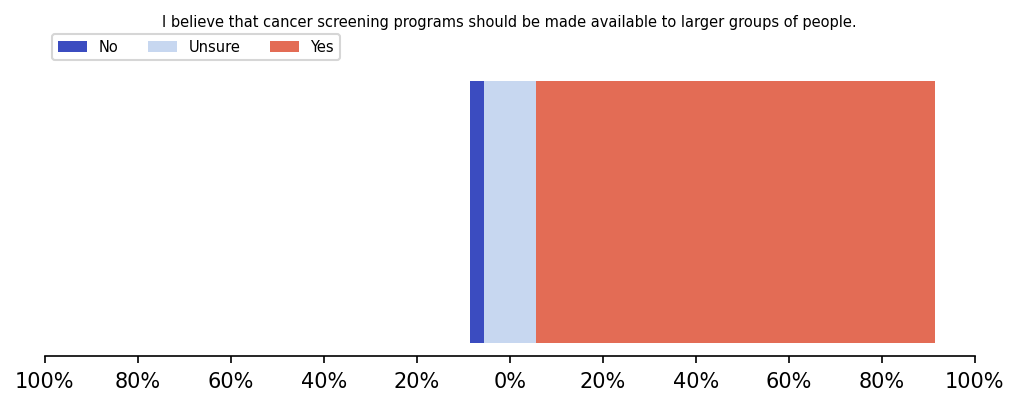


- *I have previously been refused a genetic test by a doctor or genetics specialist because I was told I was not eligible for the test.*


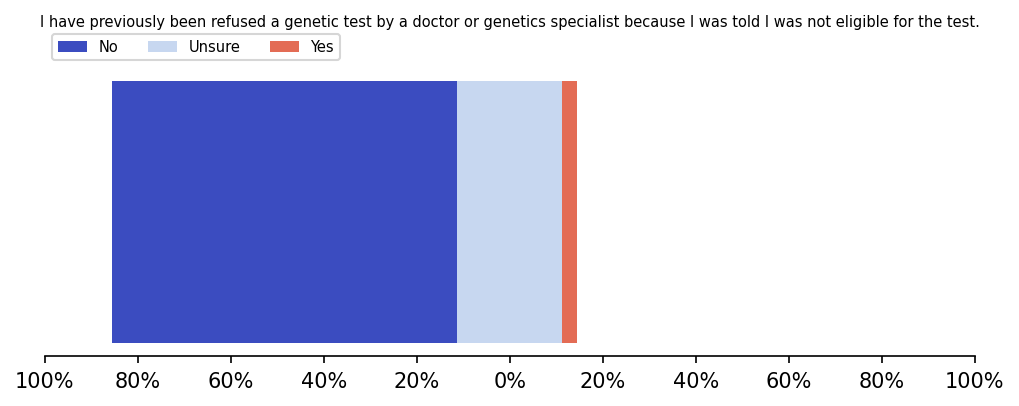


- *I have previously paid for a genetic test to be taken by a healthcare professional because I had been refused that test on the NHS.*


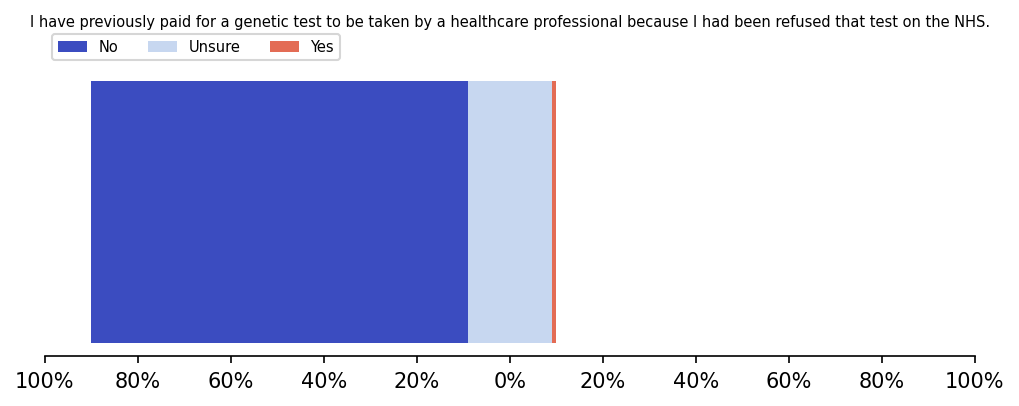


- *I have previously paid for an over-the-counter genetic test because I had been refused a genetic test by a healthcare professional on the NHS.*


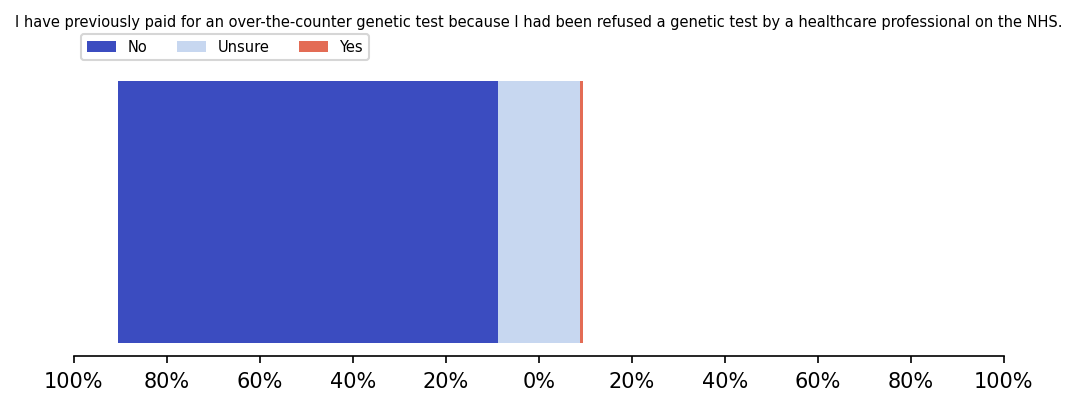


- *I have previously undertaken an over-the-counter genetic test without the involvement of a healthcare professional.*


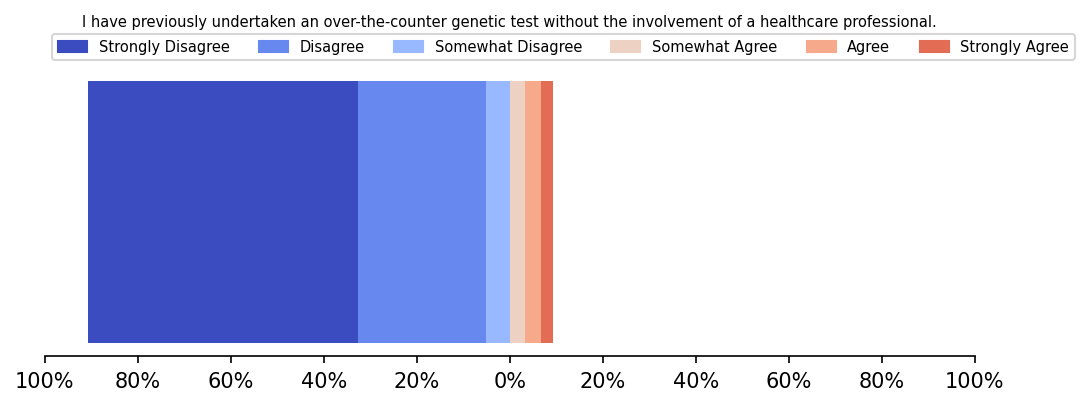


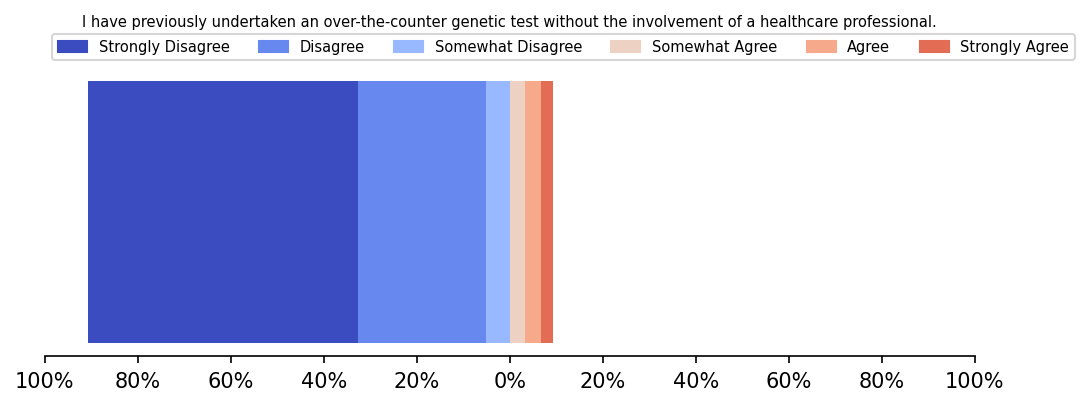


**Actions/feelings as a result of a genetic test**

- *A genetic test showing that I had a higher risk of developing a specific cancer would make me more likely to find out about new treatments for that cancer.*


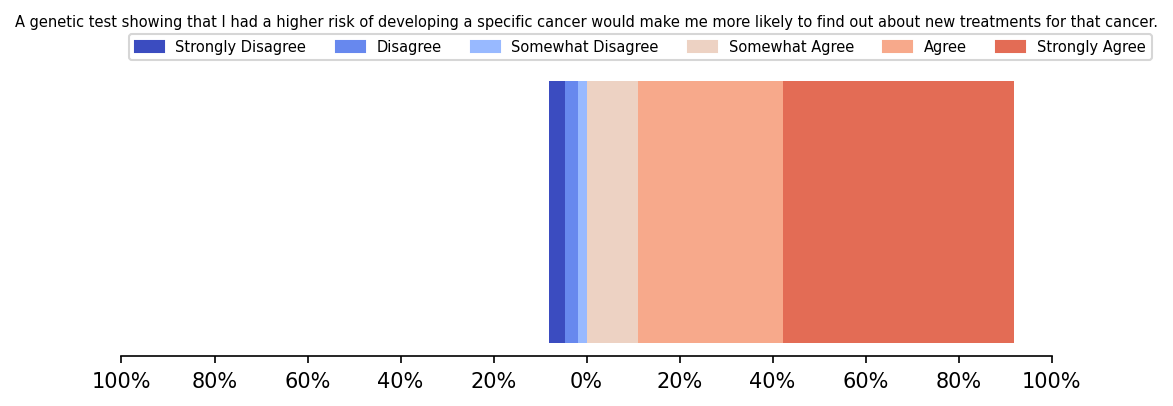


- *Treatment and prevention options for cancer are limited, so learning from a genetic test that I had an increased risk of developing cancer wouldn’t help much.*


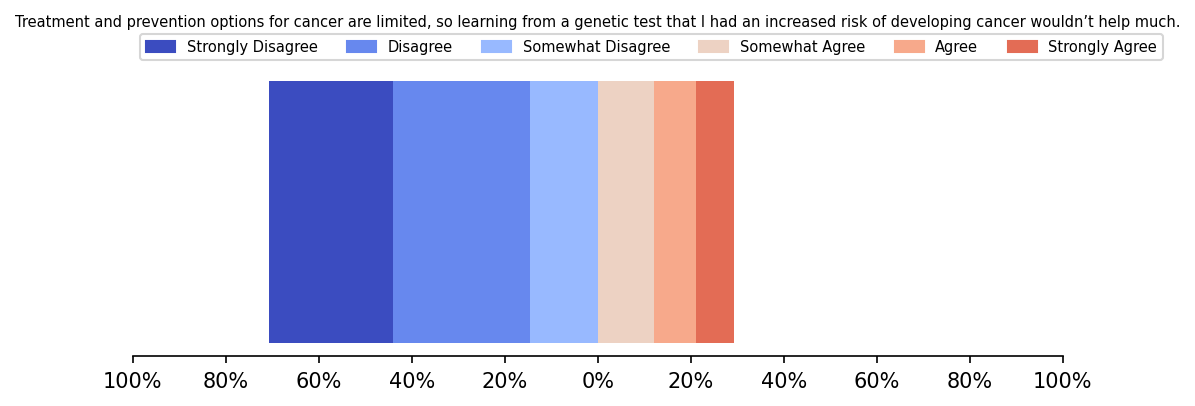


- *It would be too upsetting to learn from a genetic test that I have an increased risk of developing cancer, so I am happier not knowing.*


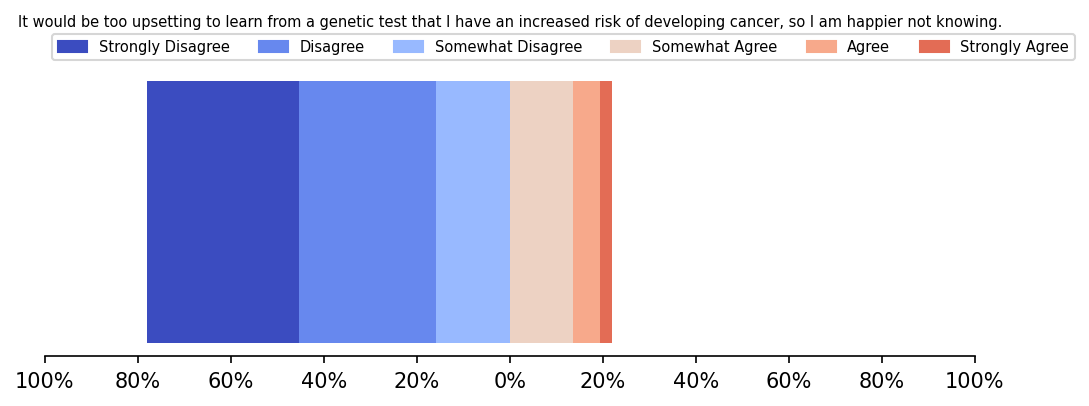


- *If I learnt from a genetic test that I had an increased risk of developing cancer, I would be concerned about my emotional reactions.*


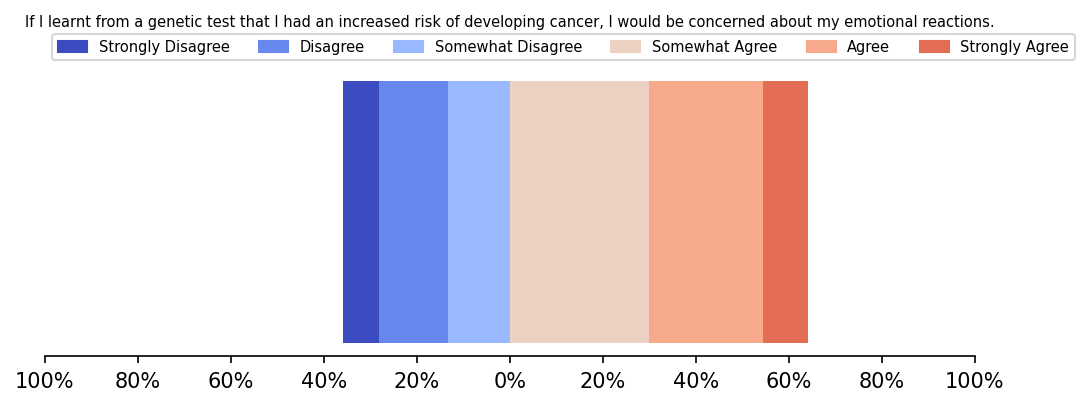


- *If I learnt from a genetic test that I had an increased risk of developing cancer, I would be concerned about my partner’s reaction.*


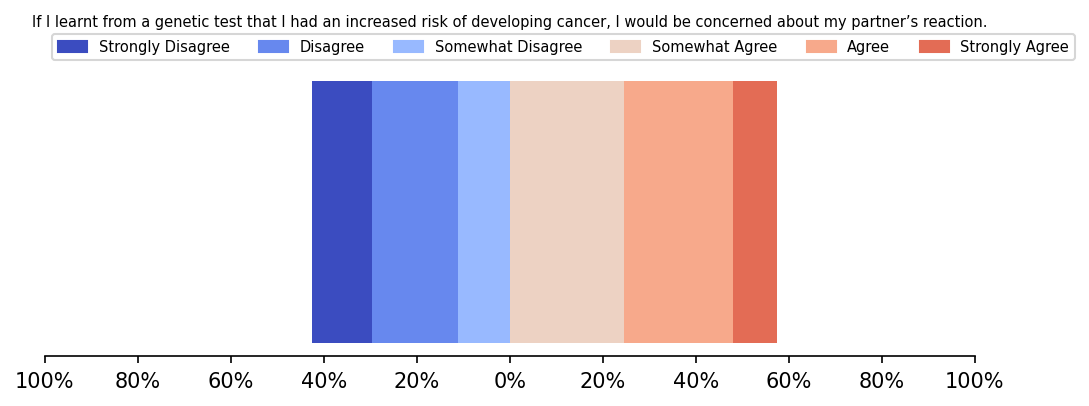


- *If I learnt from a genetic test that I had an increased risk of developing cancer, I would be concerned about my family’s reaction.*


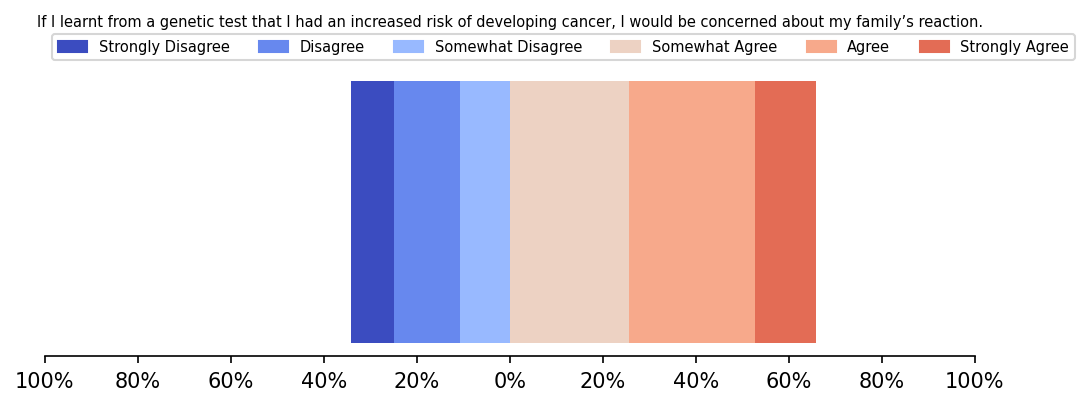


- *If I learnt from a genetic test that I had an increased risk of developing cancer, I would worry whether the test was accurate.*


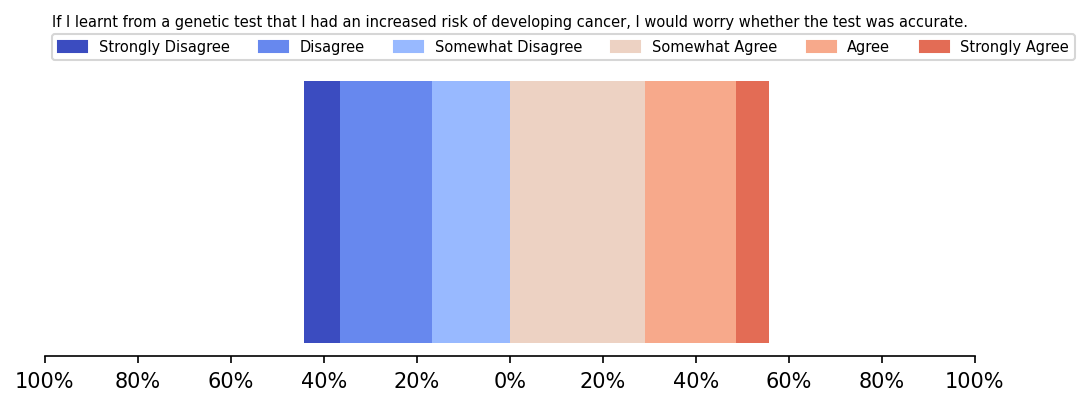


- *If I learnt from a genetic test that I had an increased risk of developing cancer, I would worry about how it would affect my health and/or life insurance.*


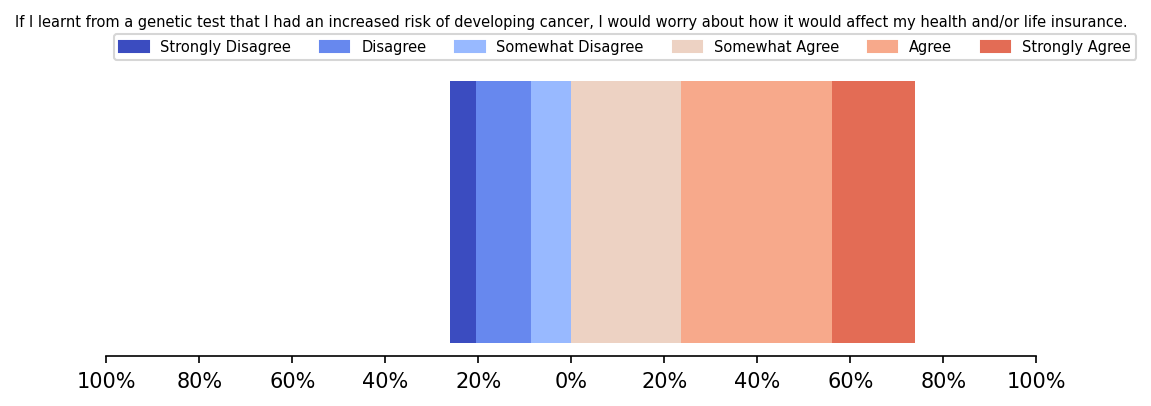


- *If I learnt from a genetic test that I had an increased risk of developing cancer, I would worry that it would affect my chances of finding a job.*


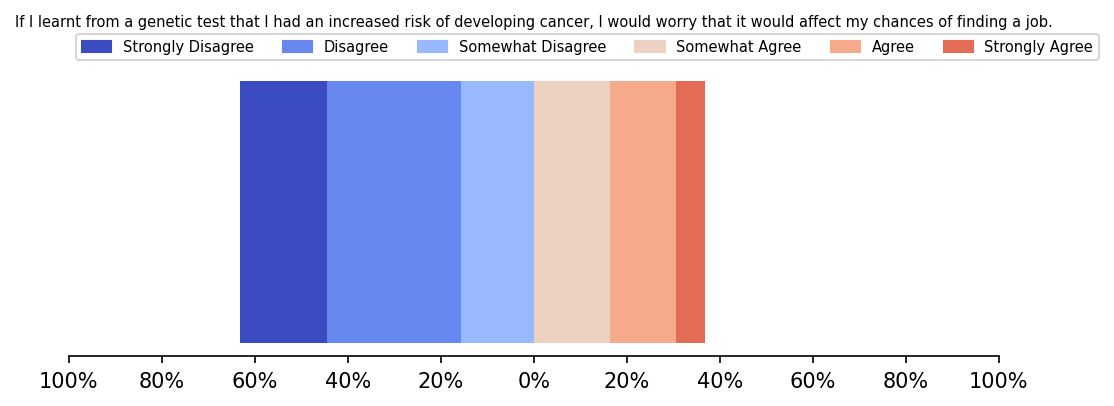


- *If I learnt from a genetic test that I had an increased risk of developing cancer, it would help me plan for the future.*


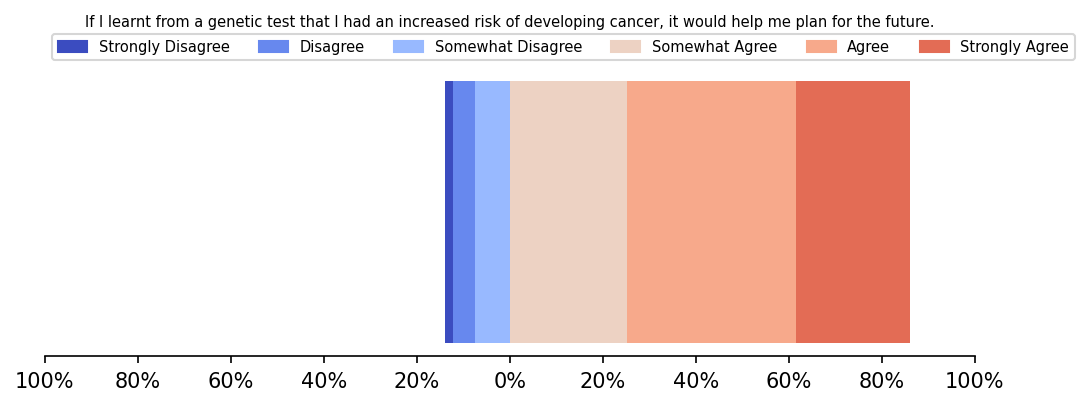


- *I would wish to undergo surgery to reduce my risk of developing cancer if my risk of developing that cancer in my lifetime was:*


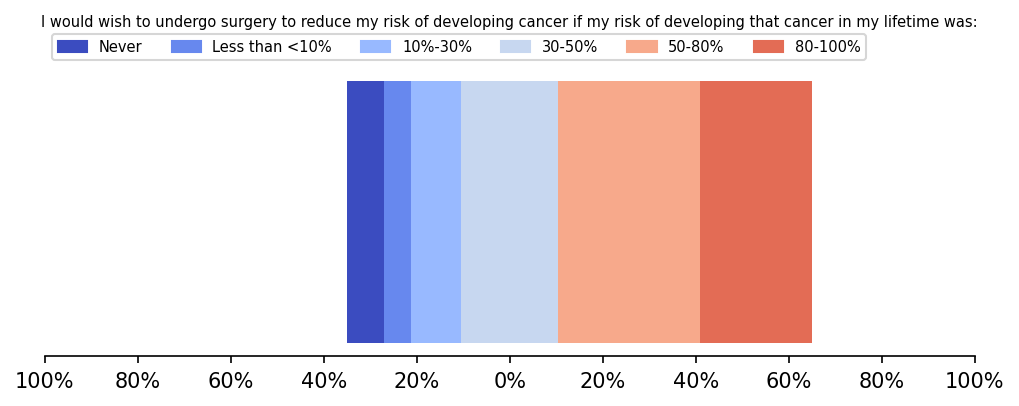


- *I would wish to take a medication to reduce my risk of developing cancer if my risk of developing that cancer in my lifetime was*


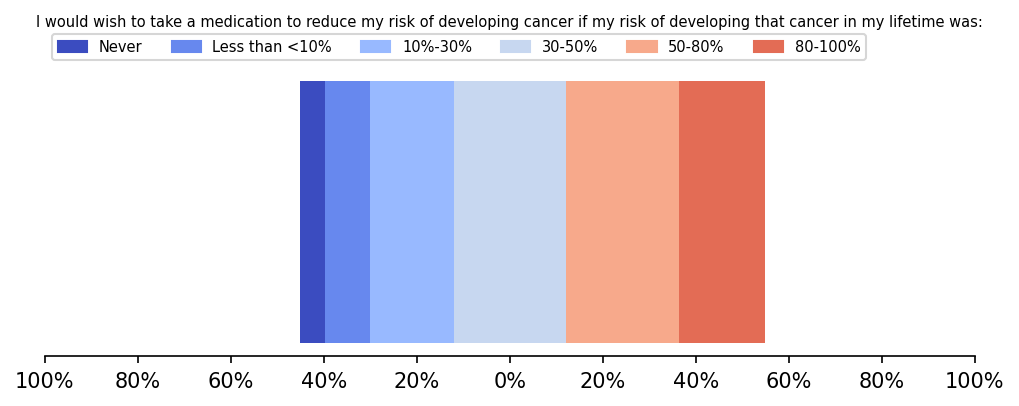


- *I would make changes to my lifestyle to reduce my risk of developing cancer if my risk of developing that cancer in my lifetime was:*


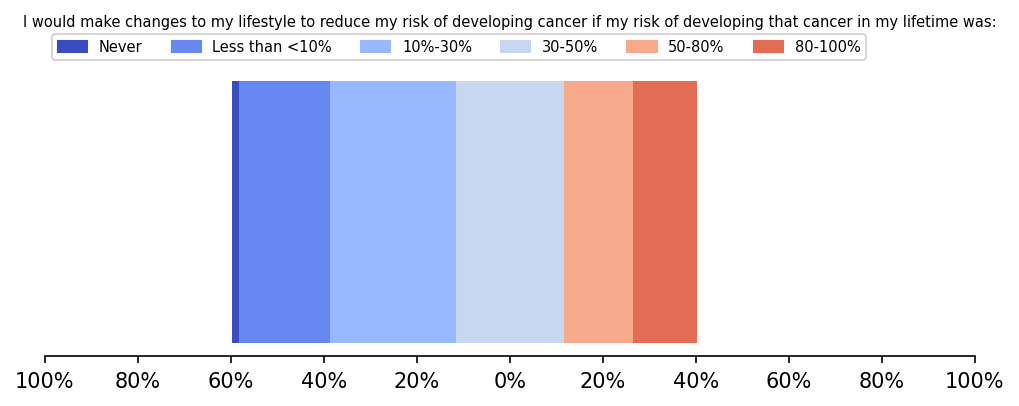


- *I would make changes to my diet to reduce my risk of developing cancer if my risk of developing that cancer in my lifetime was:*


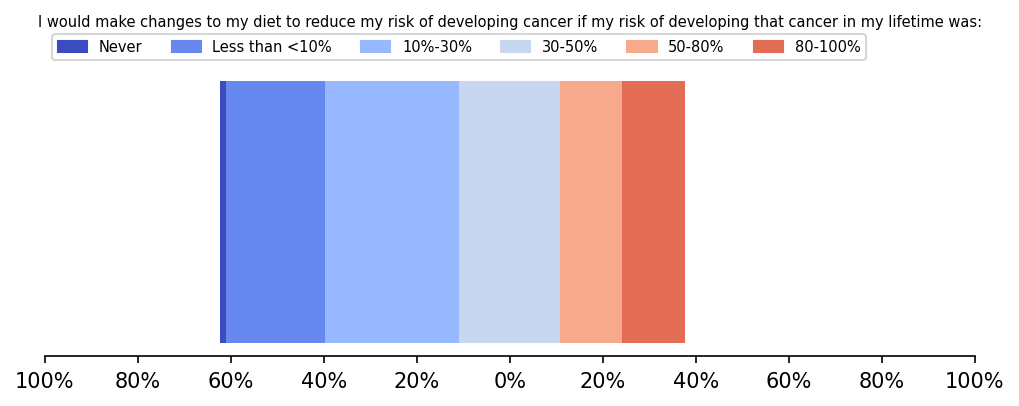


**Concerns/Apprehension around genetic testing**

- *I worry that having a genetic test might change my future.*


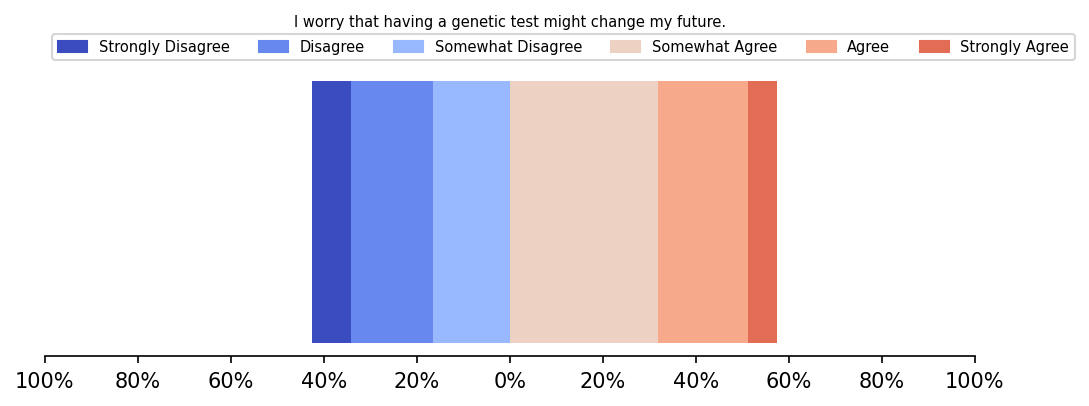


- *I do not want to know what kind of diseases I could get in the future.*


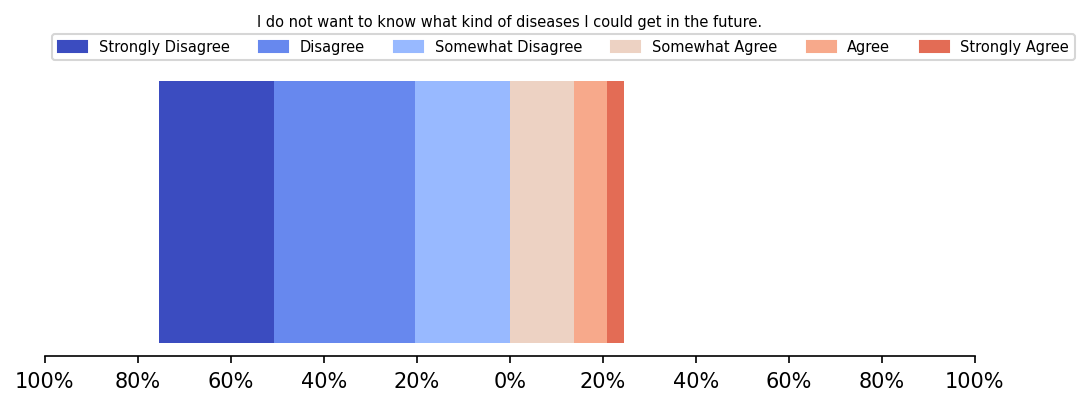


- *I am more likely to believe the results of a genetic test undertaken by my doctor than one I paid for over-the-counter at a chemist or supermarket.*


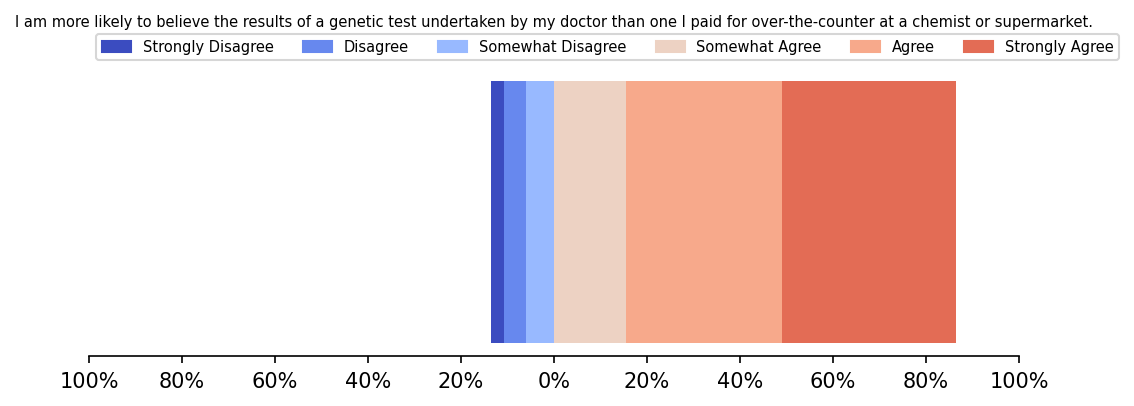


- *If a specific disease cannot be treated, I would not want to have a genetic-test that identifies a risk of developing that disease.*


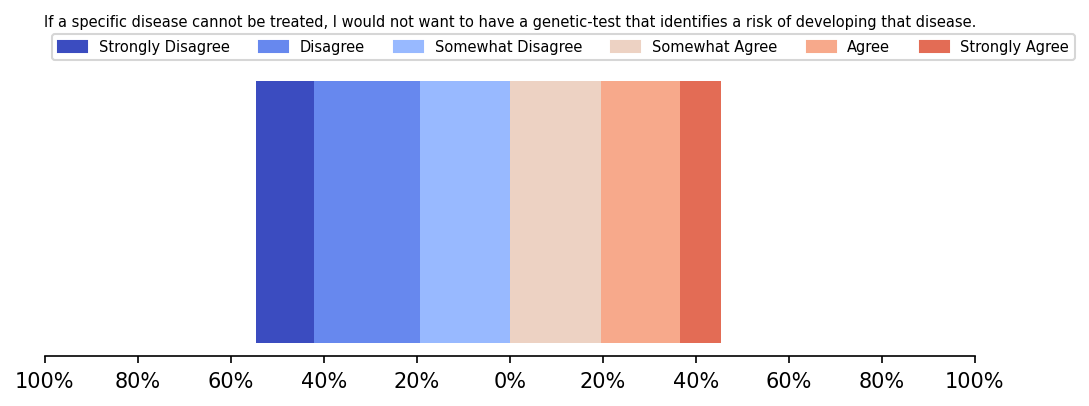


- *If I had a genetic test done I would want to be able to select what diseases it would or would not assess my risk for.*


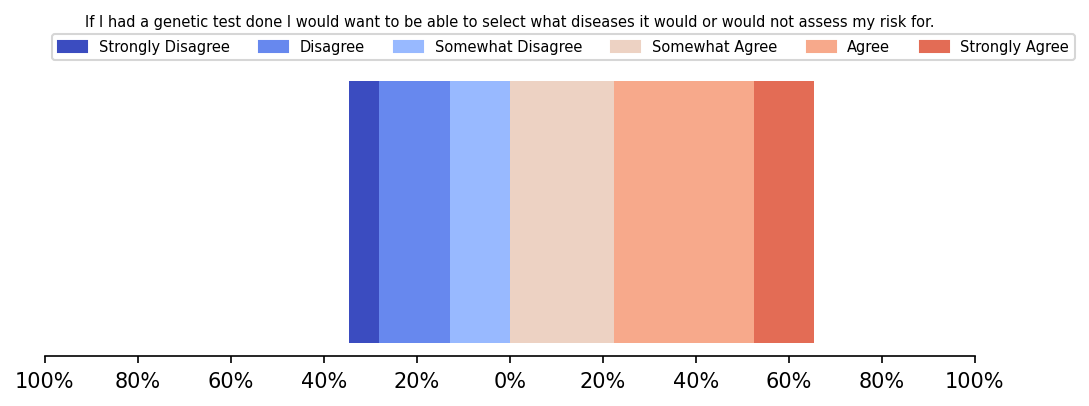


- *The idea of a genetic test frightens me.*


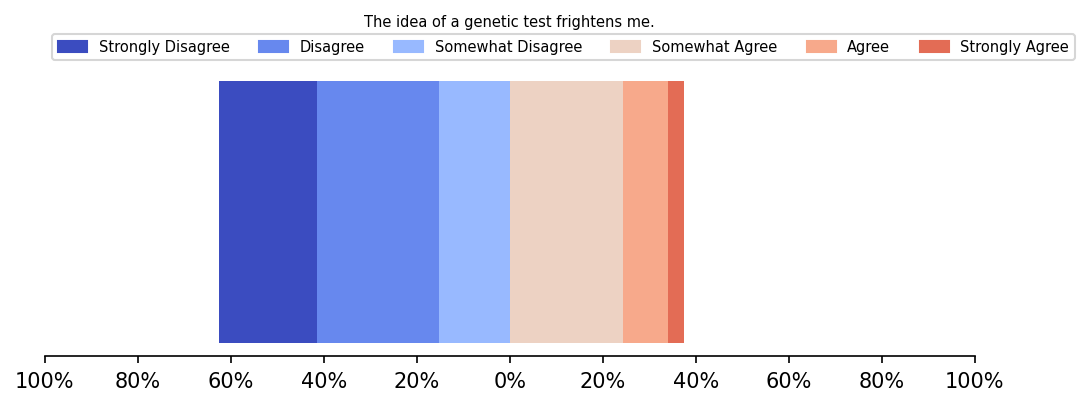


**Future usages of genetic tests**

- *If a genetics blood or saliva test became available that could accurately show whether or not I had inherited an increased risk of developing cancer, I would want to have that test.*


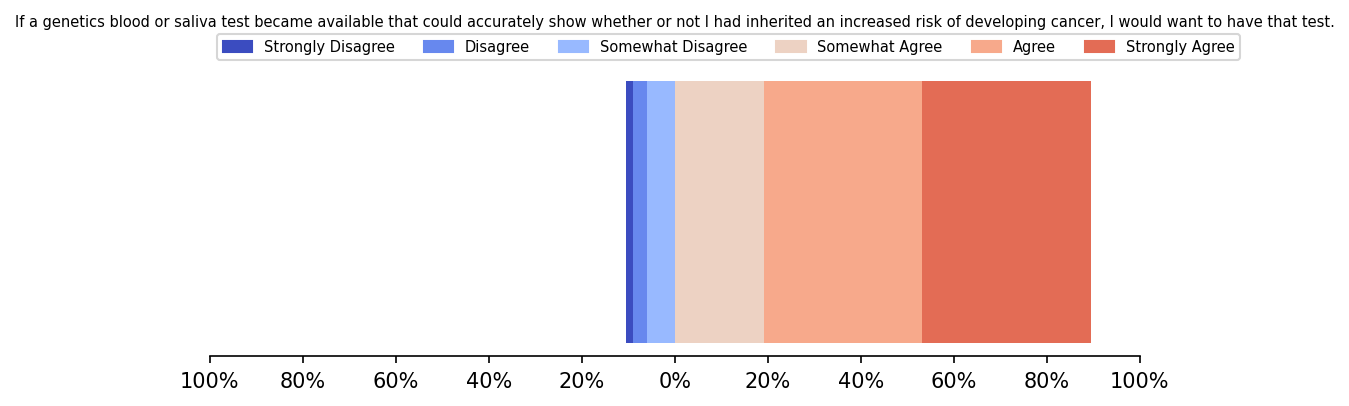


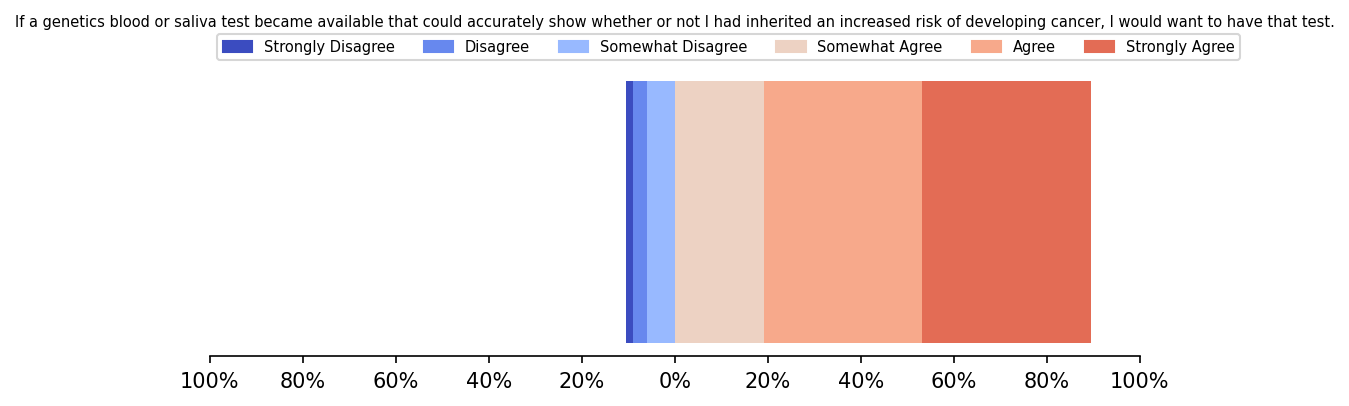


- *If a genetics blood or saliva test became available that could accurately show whether or not an individual has inherited an increased risk of developing cancer, then it should only be offered to people with a strong family history of cancer.*


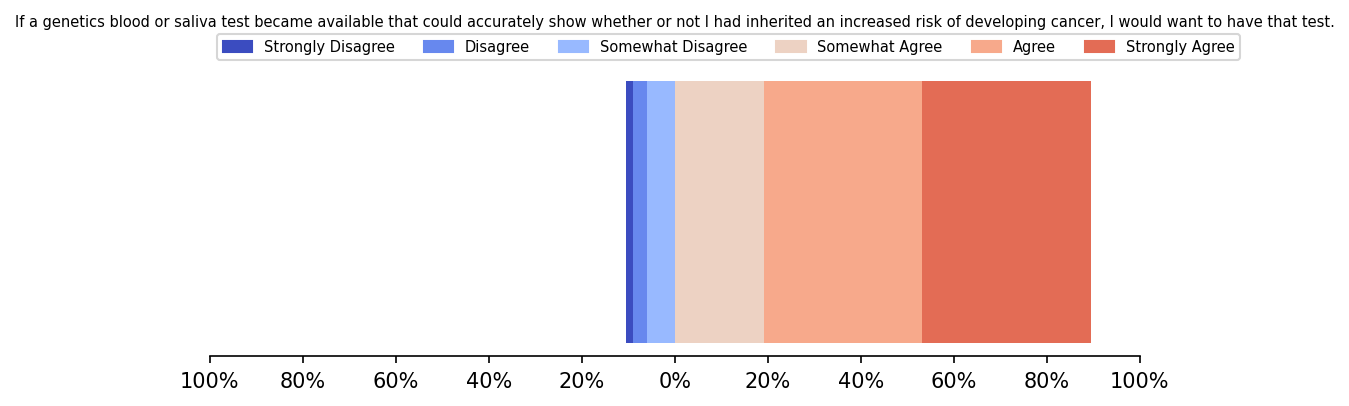


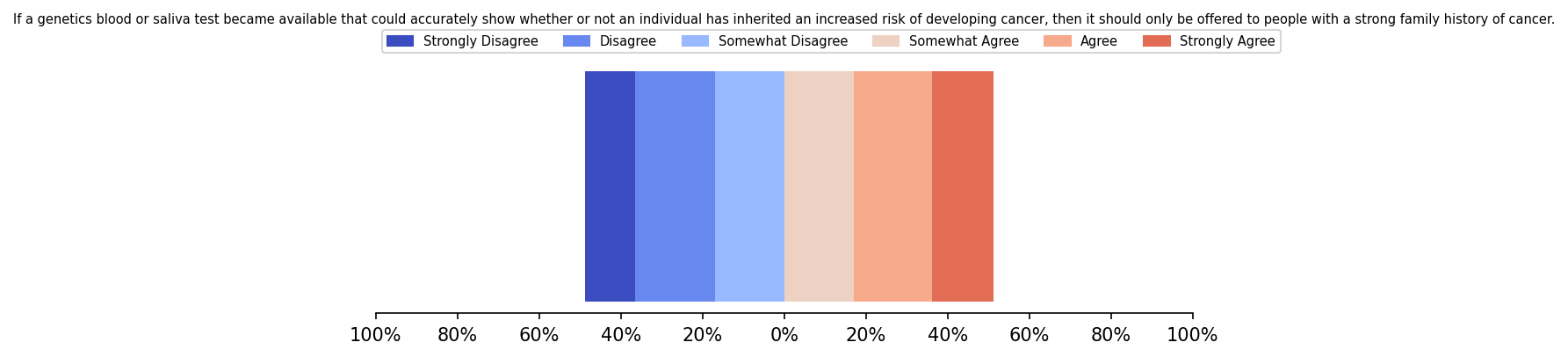


- *If a genetic test became available that could accurately show whether or not an embryo or foetus had inherited an increased risk of developing cancer, then embryo/foetal testing should be offered.*


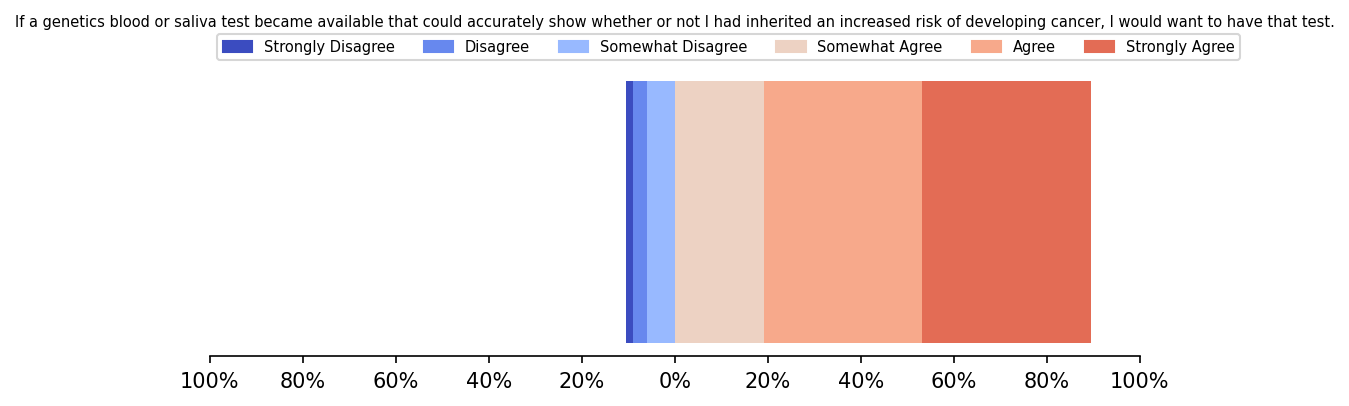


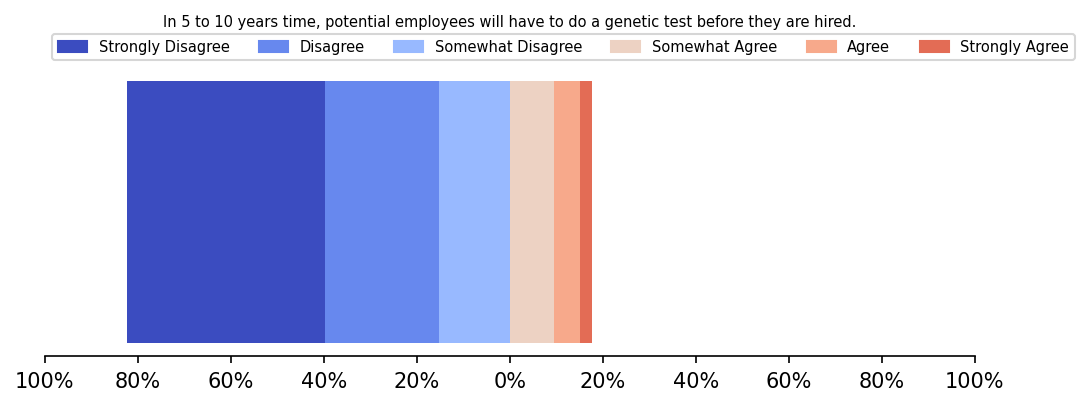


- *I would consider having a genetic test that was available over the counter at a chemist or supermarket, without the involvement of a healthcare professional.*


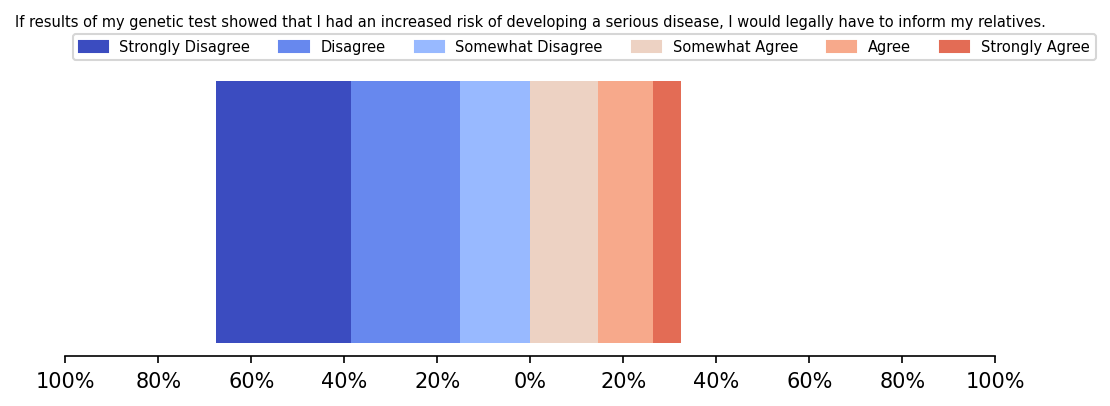


- *More people should be able to have genetic tests.*


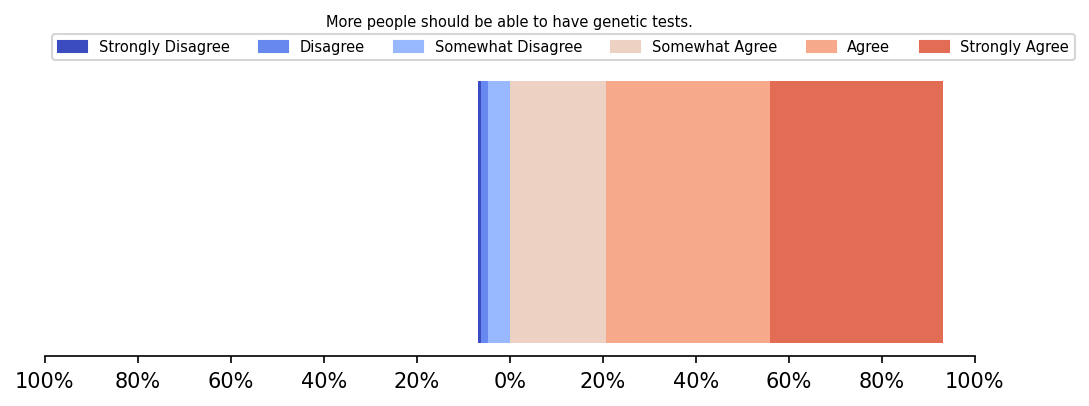


- *Genetic tests should be used to select people for cancer screening programs.*


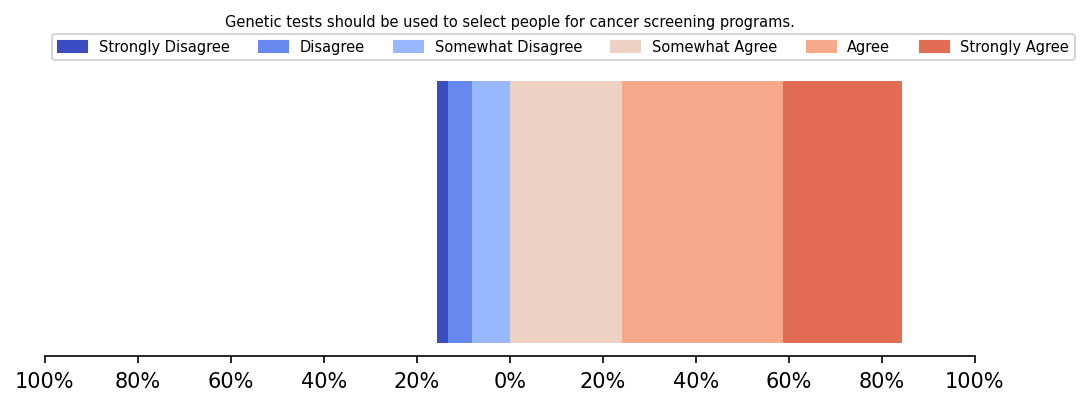


- *More money should be available for the development of genetic tests*


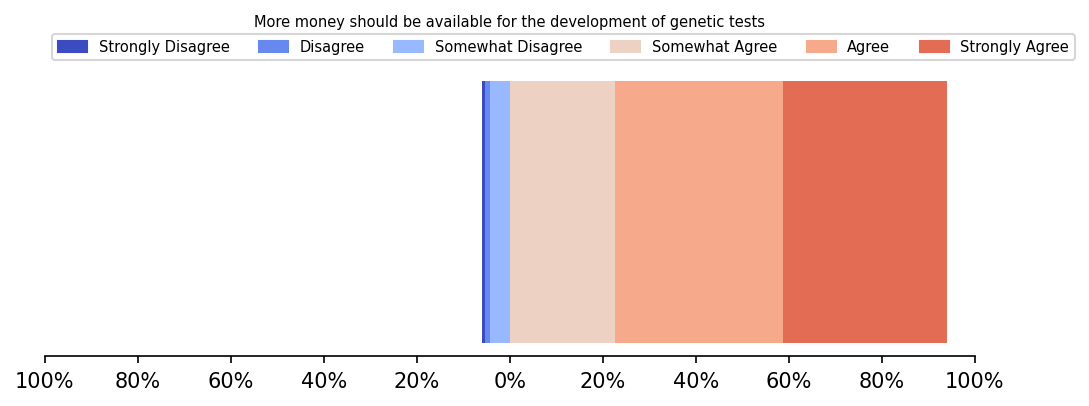


- *If I undertook a genetic test, third parties would have the right to enquire about the results of the test.*


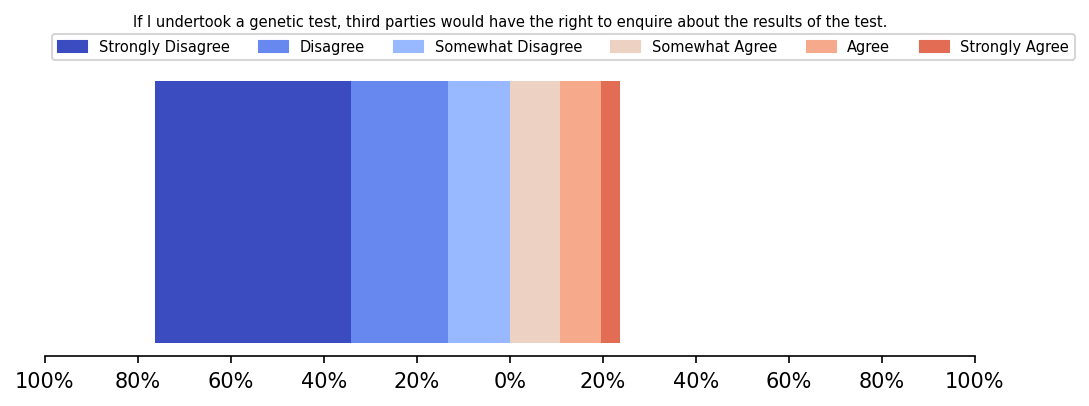


- *If I undertook a genetic test the results would be confidential.*


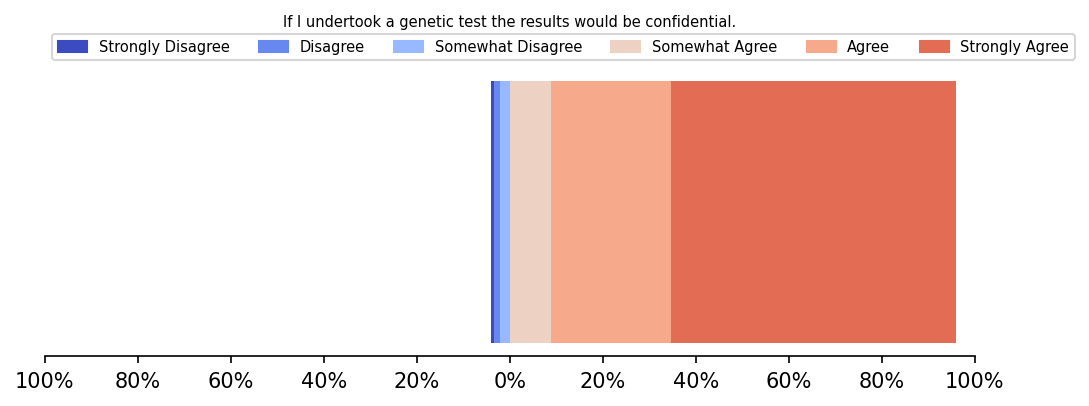


- *If results of my genetic test showed that I had an increased risk of developing a serious disease, I would legally have to inform my relatives.*


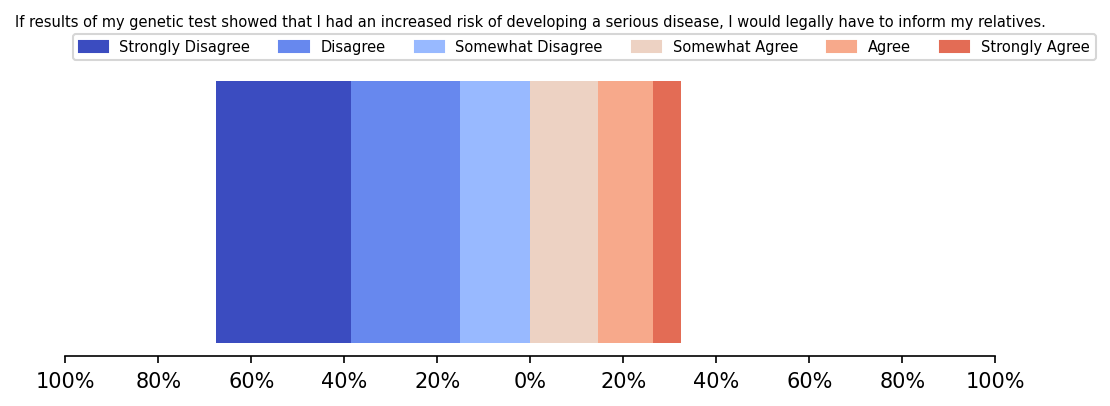


- If I have or develop a disease, I would want to know whether it was inherited if possible.


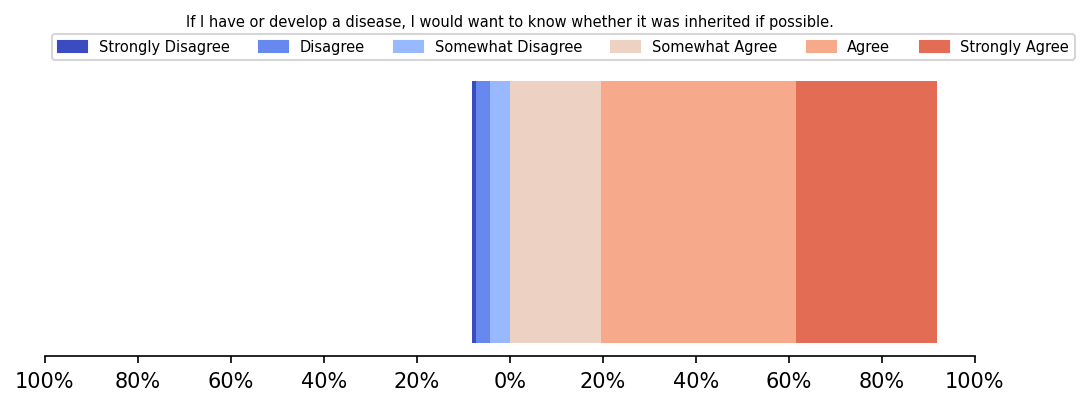


- The development of genetic research is hopeful for the treatment of diseases.


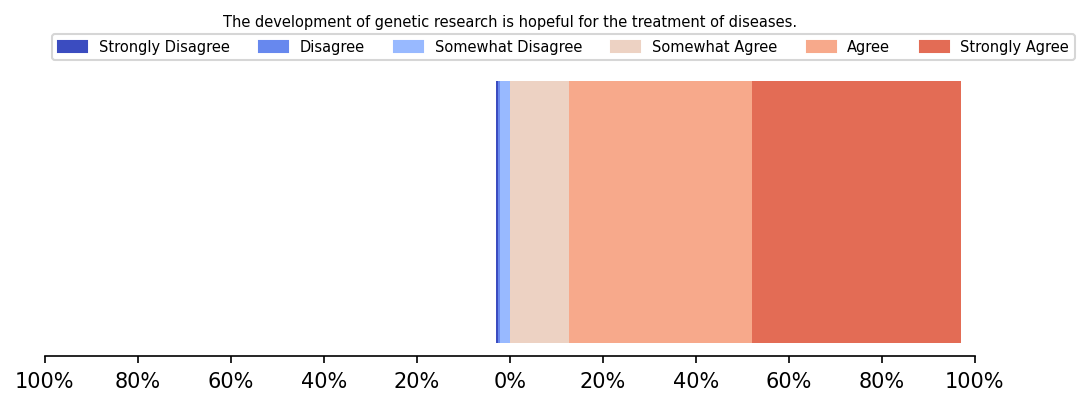


- *In 5 to 10 years time, it will be common to have a genetic test.*


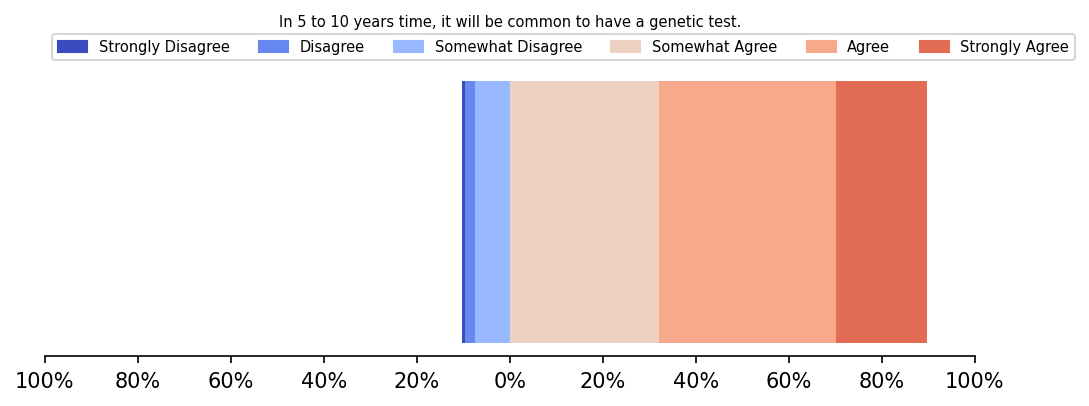


- *In 5 to 10 years time, we will all get a genetic passport.*


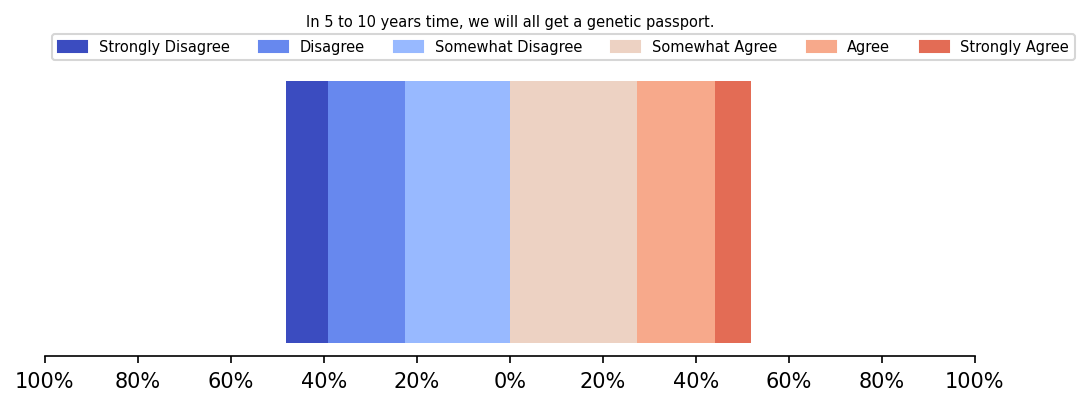


- *In 5 to 10 years time, our genetic information will be stored in computers.*


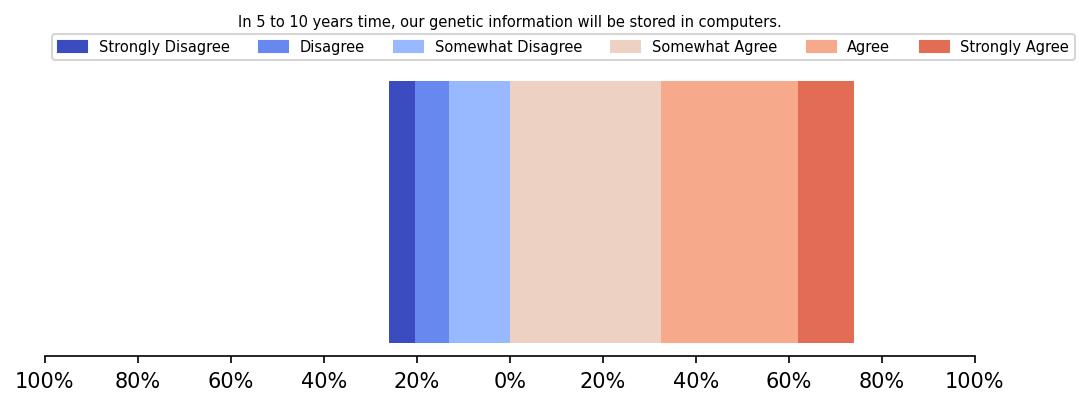


- *In 5 to 10 years time, all children will be tested at a young age to find out what diseases they are at risk of getting at later age.*


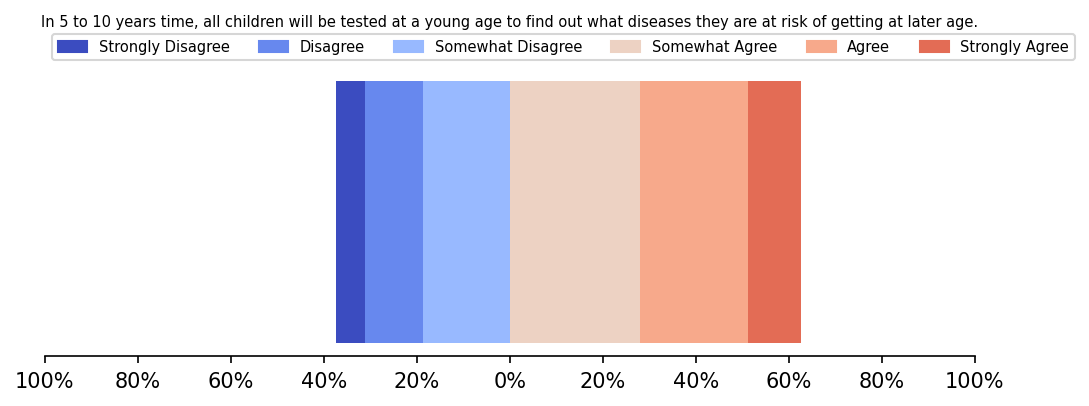


- *In 5 to 10 years time, potential employees will have to do a genetic test before they are hired.*


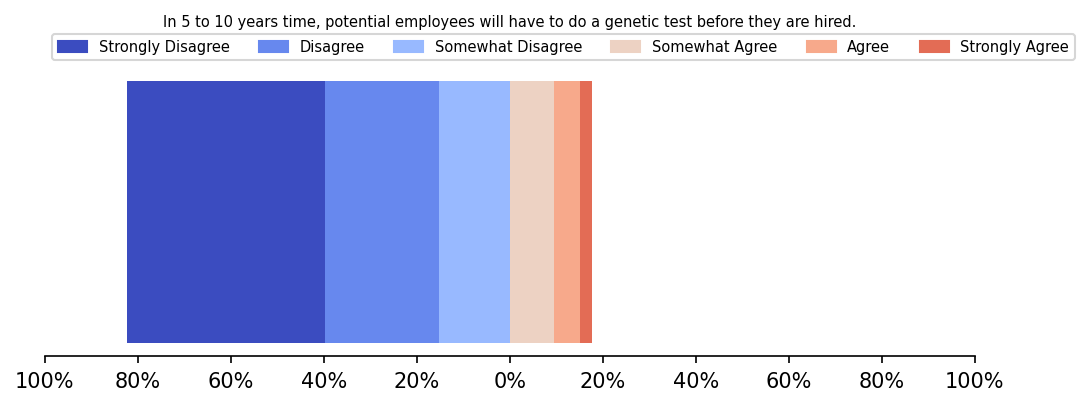


- *In 5 to 10 years time, insurance companies will ask for a genetic test before the height of the premium is set.*


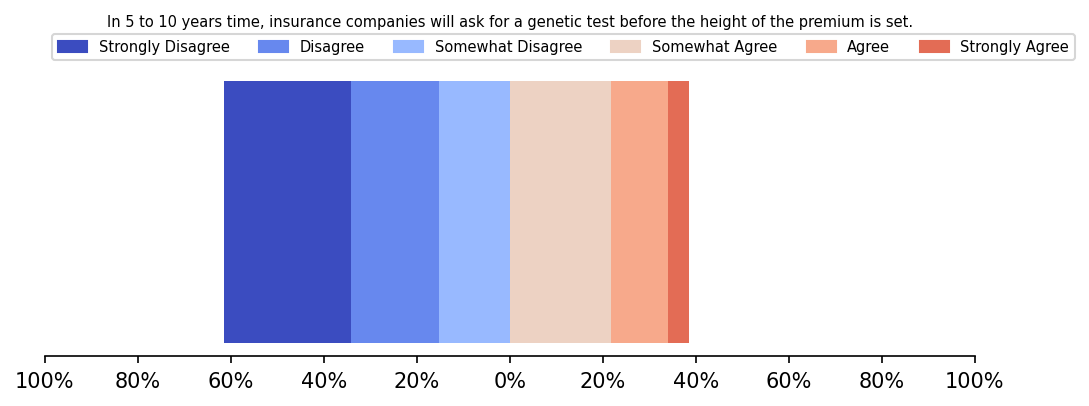


**Understanding of biology/genetics**

- *Cancer risk can be inherited.*


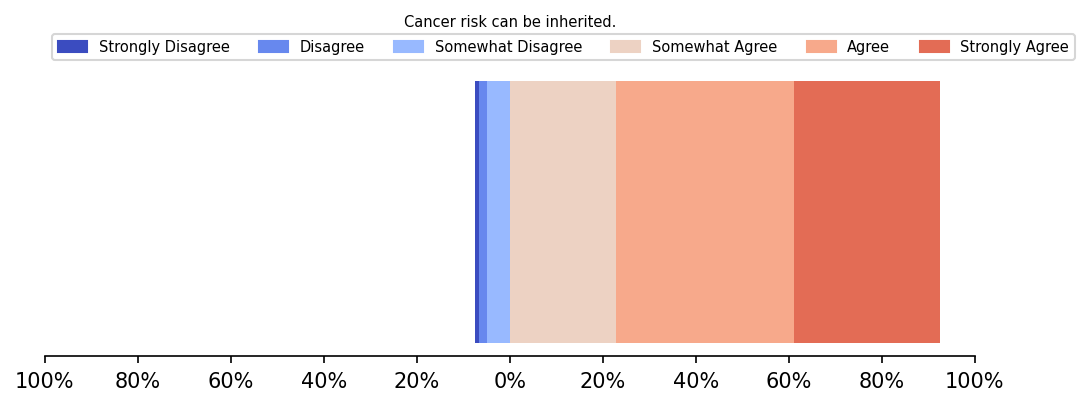


- *Genes come in pairs; one copy from each parent.*


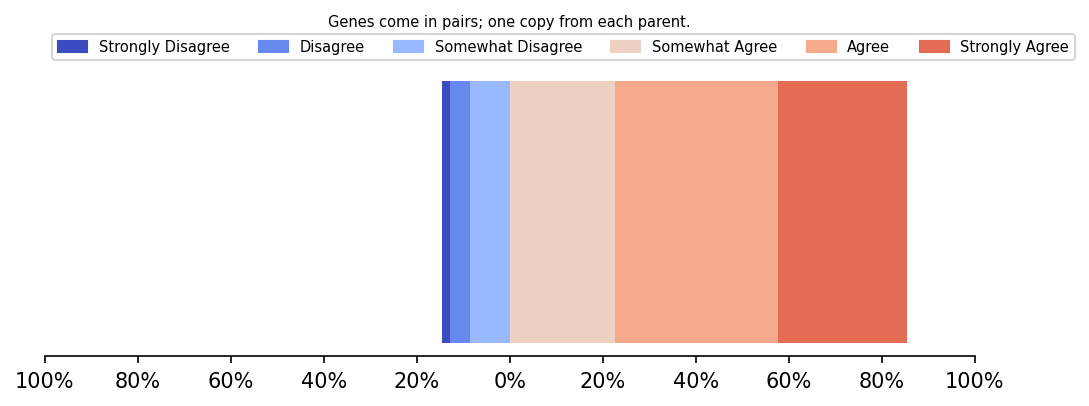


- *If I haven’t inherited any genetic abnormalities that would increase my risk of cancer, I might still develop cancer in my lifetime.*


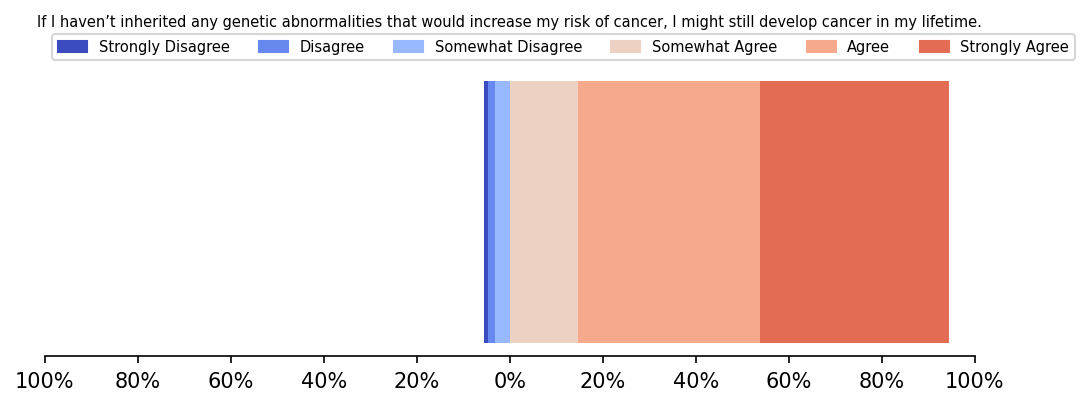


- *If I have inherited genetic abnormalities that increase my risk of cancer, this does not mean that I will definitely develop cancer.*


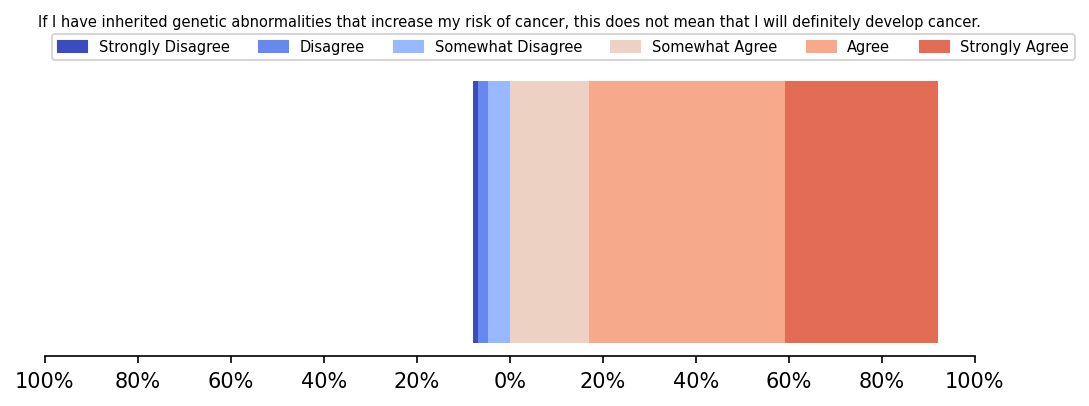


- *Hereditary genetic abnormalities linked with cancer risk, can skip generations.*


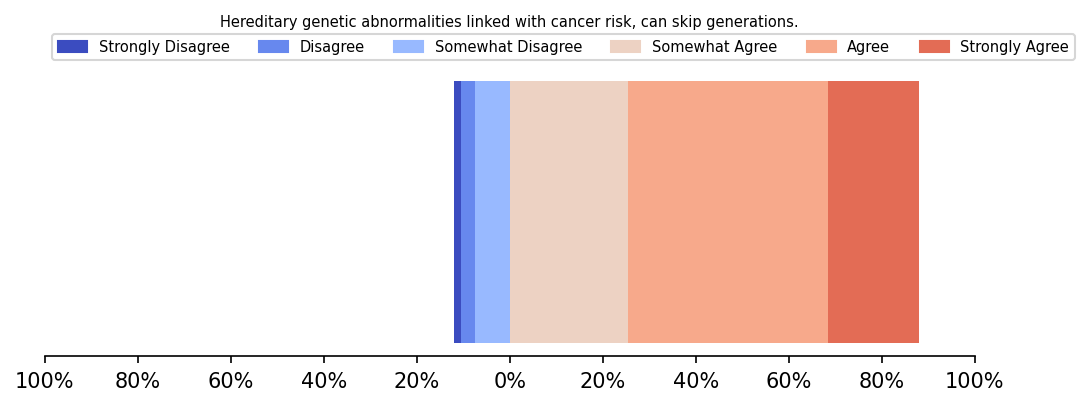


- *A gene is a molecule that controls hereditary characteristics.*


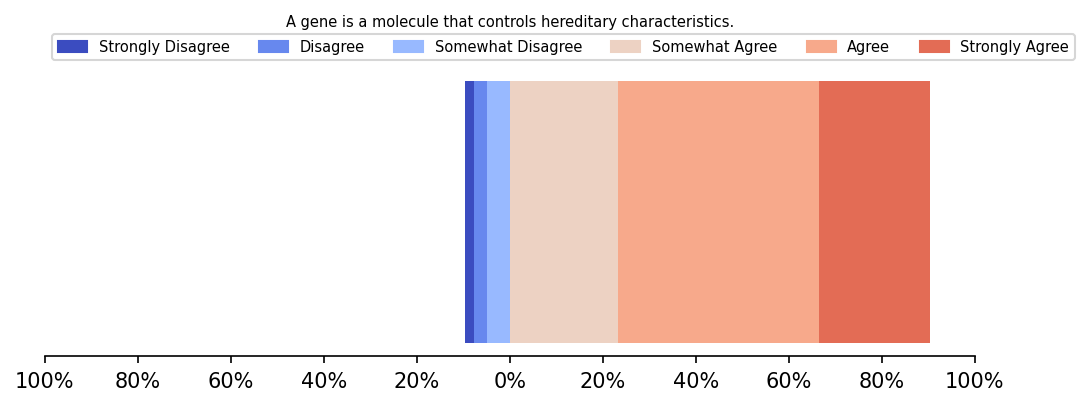


- *Genes are inside cells.*


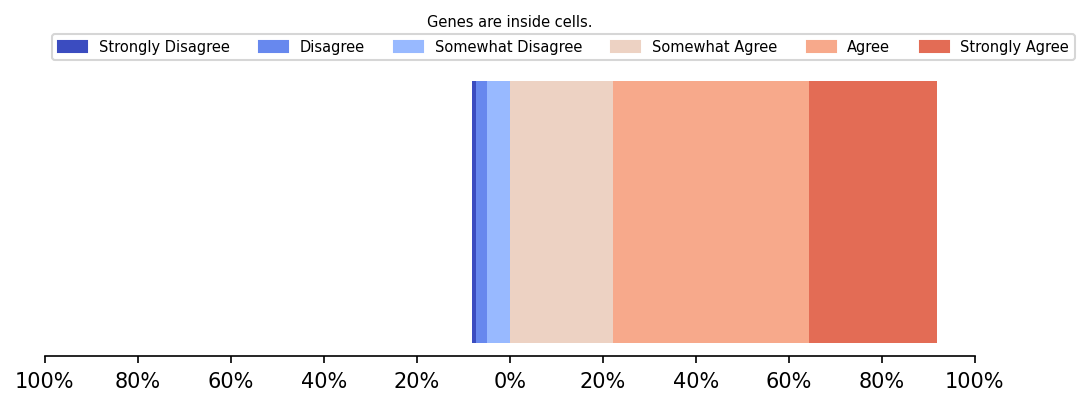


- *A gene is a piece of DNA.*


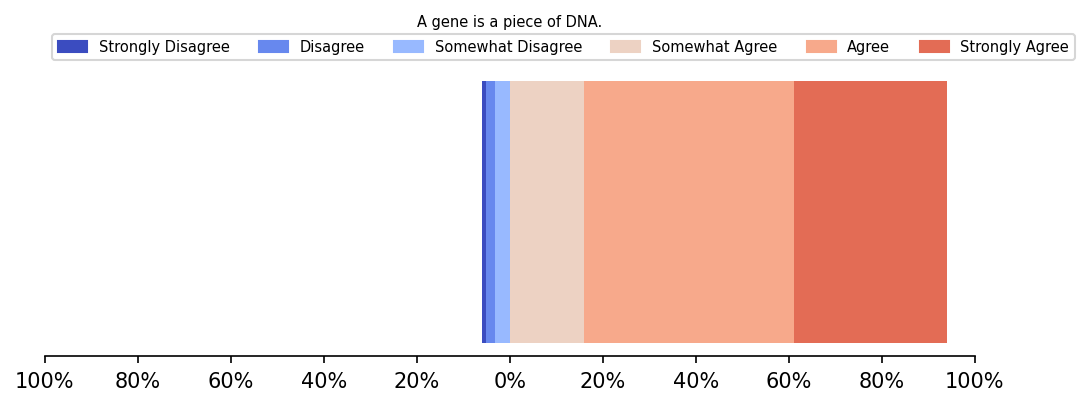


- *A gene is a part of a chromosome.*


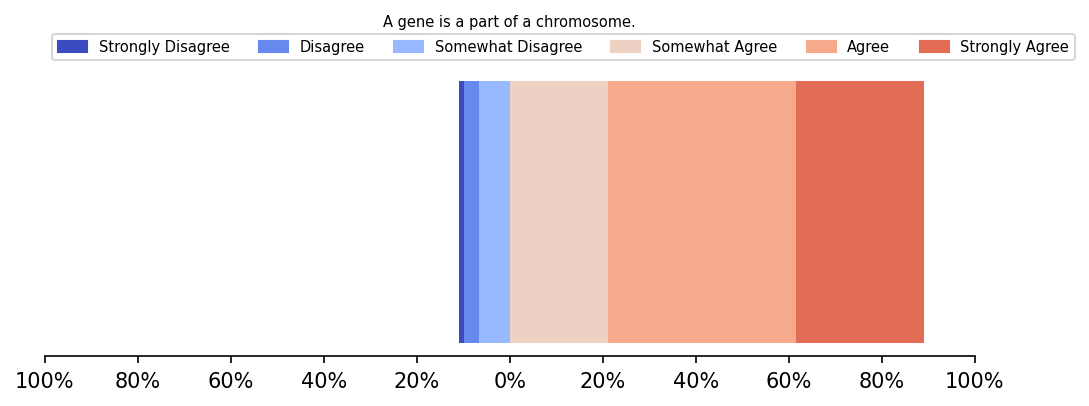


- *Different body parts include different genes.*


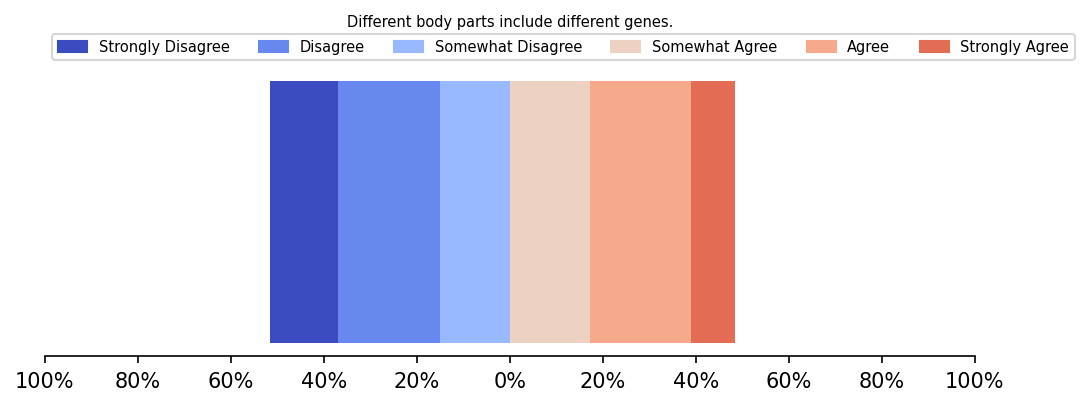


- *Genes are not susceptible to human intervention.*


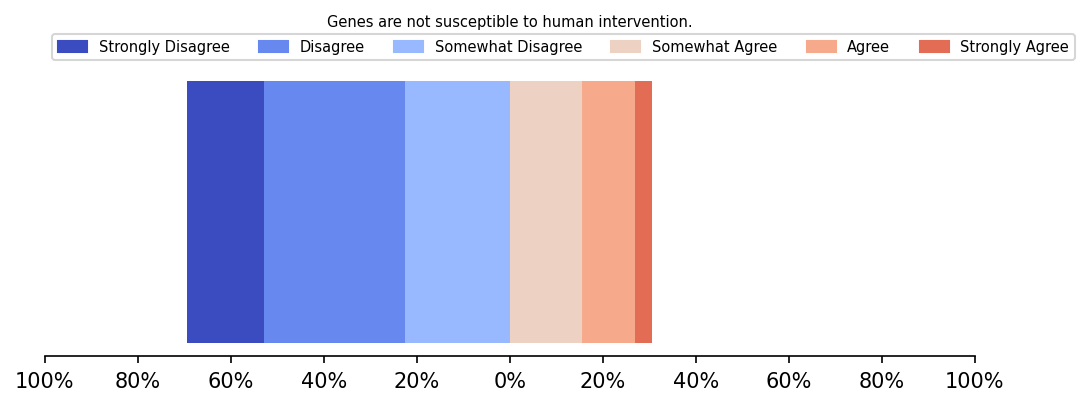


- *It has been estimated that a person has 22,000 genes.*


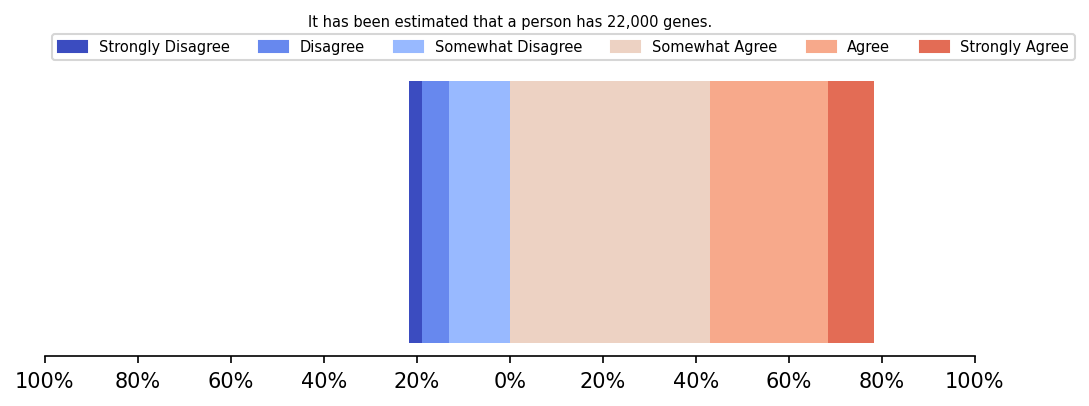


- *Healthy parents can have a child with a hereditary disease.*


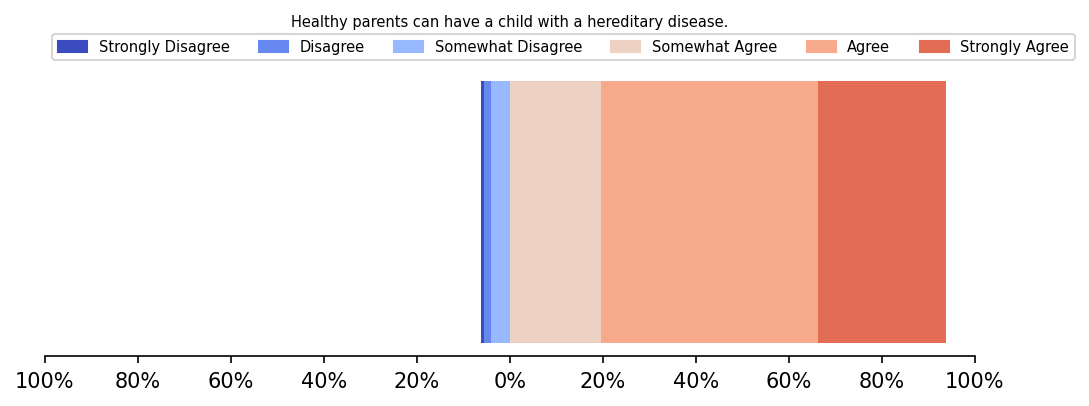


- *The onset of certain diseases is due to genes, environment, and lifestyle.*


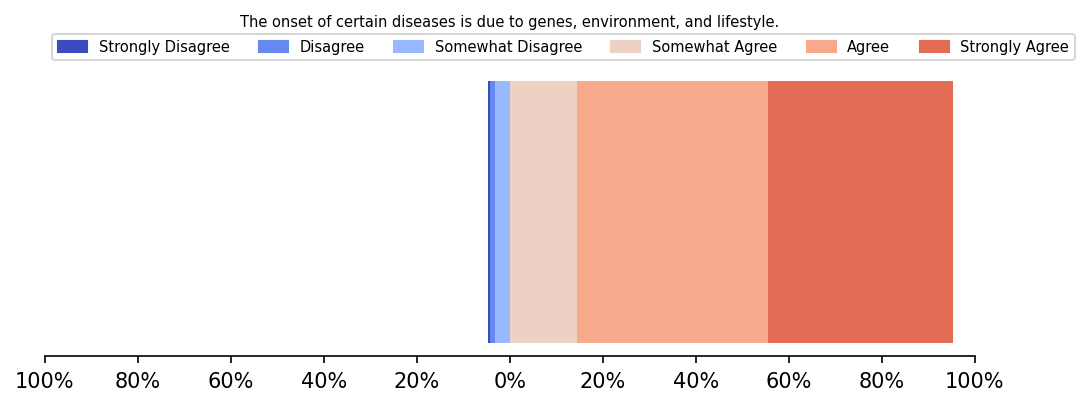


- *The carrier of a disease gene may be completely healthy.*


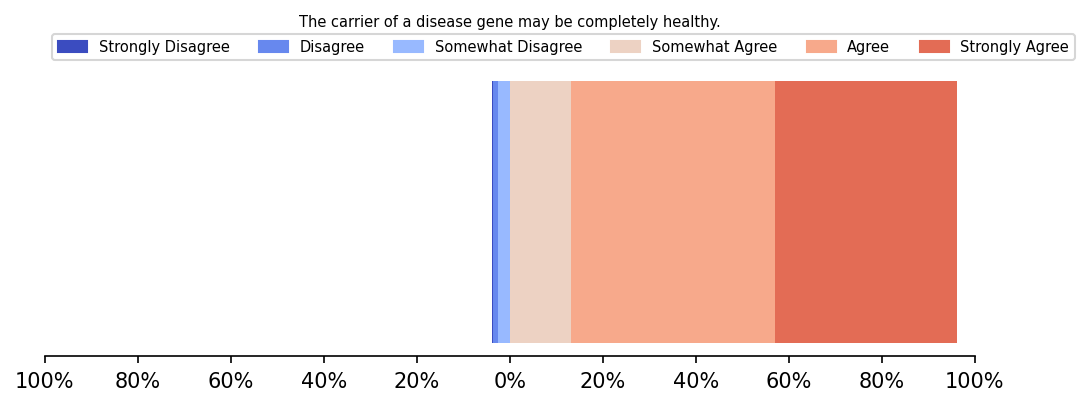


- *The child of a disease gene carrier is always also a carrier of the same disease gene.*


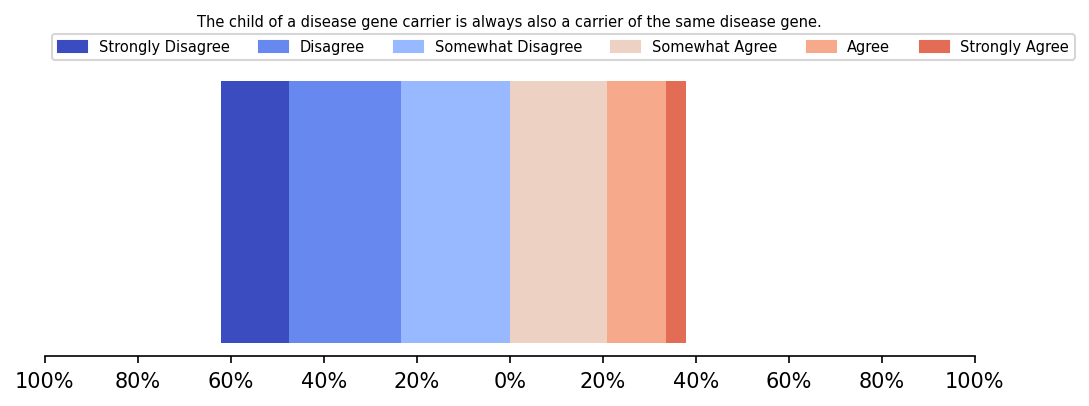


- *All serious diseases are hereditary.*


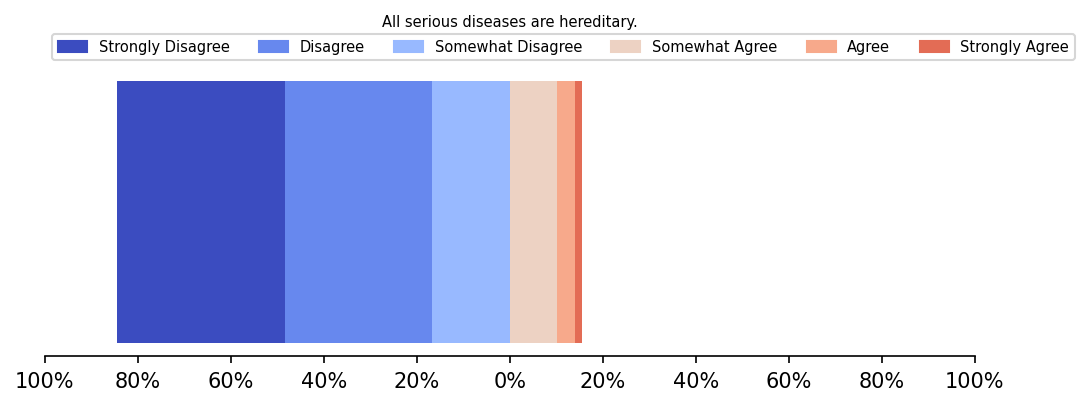

Supplement: Supplementary file 4 — Appendix 1 [file 10038_2023_1199_MOESM4_ESM.docx]
